# Supplementary material for: Patient perspectives on the usability and content validity of the assessment of burden of chronic conditions tool for post-COVID in the Netherlands: a qualitative study
Source: BMJ Open. 2025 Dec 11;15(12):e109201. doi: 10.1136/bmjopen-2025-109201 (PMC12706107; doi:10.1136/bmjopen-2025-109201)
Supplement: Supplementary data [file bmjopen-15-12-s001.pdf]

APPENDIX 1: QUESTIONNAIRE IN THE ABCC-TOOL

ENGLISH VERSION

(Note: this is an unvalidated translation from Dutch to English specifically for this paper)

Dear Sir/Madam,
With this questionnaire, we would like to get an impression of how you are doing. During consultation with your healthcare provider, you can talk about the topics that are important to you.
These questions are related to the chronic condition(s) for which you are visiting the healthcare provider.

What were you suffering from?

	In the past week, how often ….	0 Never	1 Hardly ever	2 A few times	3 Several times	4 Many times	5 A great many times	6 Almost all the time
1	did you suffer from **fatigue**?	□	□	□	□	□	□	□
2	did you have a poor **night's rest**?	□	□	□	□	□	□	□
3	did you suffer from sadness, fear, frustration, shame or other **unpleasant feelings**?	□	□	□	□	□	□	□
4	did you suffer **adjusting your life** (e.g. planning of activities, get enough exercise, eating healthy)?	□	□	□	□	□	□	□

	In the past 2 weeks, to what extend …	0 Not at all	1 Very slightly	2 Slightly	3 Moderate	4 Very	5 Extremely	6 Totally
5	did you suffer from a **decreased condition**?	□	□	□	□	□	□	□
6	did you suffer from **pain/ difficulties with breathing**?	□	□	□	□	□	□	□
7	did you suffer from **coughing**?	□	□	□	□	□	□	□
8	did you suffer from **pain on the chest** or **a feeling of pressure on the chest**?	□	□	□	□	□	□	□
9	did you suffer from **palpitations**?	□	□	□	□	□	□	□
10	did you suffer from a **strange taste or smell, or tasted/smelled nothing**?	□	□	□	□	□	□	□
11	did you suffer from **earache** or **tinnitus**?	□	□	□	□	□	□	□
12	did you suffer from a **lump in your throat** or a **sore throat**?	□	□	□	□	□	□	□
13	did you suffer from problems with **chewing and/or swallowing**?	□	□	□	□	□	□	□
14	did you suffer from **heavy arms or legs**?	□	□	□	□	□	□	□
15	did you suffer from **joint and/or muscle pain**?	□	□	□	□	□	□	□
16	did you suffer from **tingling in your hands, feet, arms or legs**?	□	□	□	□	□	□	□
17	did you suffer from **a headache**?	□	□	□	□	□	□	□
18	did you suffer from **dizziness**?	□	□	□	□	□	□	□
19	did you suffer from problems with **concentration and/or memory**?	□	□	□	□	□	□	□

In the past 2 weeks, to what extend …		0<br>Not at all	1<br>Very slightly	2<br>Slightly	3<br>Moderate	4<br>Very	5<br>Extremely	6<br>Totally
20	did you feel **overstimulated**?	□	□	□	□	□	□	□
21	did you suffer from **bad memories, flashbacks or nightmares**?	□	□	□	□	□	□	□
22	did you suffer from **blurred and/or double vision, dry or painful eyes**?	□	□	□	□	□	□	□
23	did you suffer from **abdominal complaints**?	□	□	□	□	□	□	□
24	did you suffer from **feeling hot and cold alternately** or did you have a **fever**?	□	□	□	□	□	□	□
25	did you suffer from **dry or sore skin**?	□	□	□	□	□	□	□
26	did you suffer from **hair loss**?	□	□	□	□	□	□	□
In the past 2 weeks, …		Not	2-3 hours	4-10 hours	11-13 hours	14-24 hours	24-48 hours	Longer than 48 hours
27	if you **felt worse after exercising**, how long did this last?	□	□	□	□	□	□	□
28	did you suffer from **any complaints other than those mentioned above**?	I suffered from:						

In what did you feel limited?

In the past week, to what extend …		0<br>Not at all	1<br>Very slightly	2<br>Slightly	3<br>Moderate	4<br>Very	5<br>Extremely	6<br>Totally
29	were you limited in **strenuous physical** activities (such as climbing stairs, hurrying, doing sports)?	□	□	□	□	□	□	□
30	were you limited in **moderate physical** activities (such as walking, housework, carrying things)?	□	□	□	□	□	□	□
31	were you limited in **daily activities** at home (such as dressing, washing yourself)	□	□	□	□	□	□	□
32	were you limited in your **work** (paid word, unpaid work, volunteer work)?	□	□	□	□	□	□	□
33	were you limited in **moving around outside your home**?	□	□	□	□	□	□	□
34	Were you limited in your **social activities** (visiting friends and family, daytrips)?	□	□	□	□	□	□	□
35	had your condition a negative impact on your **relations with others**?	□	□	□	□	□	□	□
36	did you have any difficulty with **intimacy or sexuality**?	□	□	□	□	□	□	□
37	did you **worry about your future**?	□	□	□	□	□	□	□

How was your lifestyle?

38	In the past week, how many days have you had moderately intense **physical exercise** for 30 minutes or more?<br>E.g. walking or cycling in a fast pace<br>It may also be a minimum of 3x10 minutes	□ 0 days<br>□ 1-2 days<br>□ 3-4 days<br>□ 5 days or more
39	How many glasses of **alcohol** did you drink in the past week?	.... glasses per week
40	Do you **smoke** or have you smoked?	□ Yes. In the last week, how many (shag) cigarettes have you smoked on average per day? ____________<br>□ Previously. Stopped smoking since: _____ (month) / _____ (year)<br>□ Never
41	What is your **weight**?	.... kg
42	What is your **height**?	.... cm
43	In the past week, how many days did you **eat healthy**?	□ 0 days<br>□ 1-2 days<br>□ 3-4 days<br>□ 5 days or more
44	Is there anything else you would like to **discuss** or would like to receive more **information** about?<br>____<br>____	

DUTCH VERSION

Beste meneer/mevrouw,
Met deze vragenlijst willen we samen met u in kaart brengen hoe het met u gaat.
Deze vragen hebben te maken met de chronische aandoening(en) waarvoor u bij de zorgverlener komt.

Waar had u last van?

	In de afgelopen week, hoe vaak …	0<br>Nooit	1<br>Zelden	2<br>Af en toe	3<br>Regel-matig	4<br>Heel vaak	5<br>Meestal	6<br>Altijd
1	had u last van **vermoeidheid**?	☐	☐	☐	☐	☐	☐	☐
2	had u een slechte **nachtrust**?	☐	☐	☐	☐	☐	☐	☐
3	had u last van somberheid, angst, frustratie, schaamte of andere **vervelende gevoelens**?	☐	☐	☐	☐	☐	☐	☐
4	had u **moeite om uw leven aan te passen** (bijv. plannen van activiteiten, voldoende beweging, gezond eten)?	☐	☐	☐	☐	☐	☐	☐
	In de afgelopen 2 weken, in welke mate …	0<br>Hele-maal niet	1<br>Heel weinig	2<br>Een beetje	3<br>Tamelijk	4<br>Erg	5<br>Heel erg	6<br>Volledig
5	had u last van een **verminderde conditie**?	☐	☐	☐	☐	☐	☐	☐
6	had u last van **pijn of moeite met ademhalen**?	☐	☐	☐	☐	☐	☐	☐
7	had u last van **hoesten**?	☐	☐	☐	☐	☐	☐	☐
8	had u last van **pijn op de borst of een drukkend gevoel op de borst**?	☐	☐	☐	☐	☐	☐	☐
9	had u last van **hartkloppingen**?	☐	☐	☐	☐	☐	☐	☐
10	had u last van een **vreemde smaak of geur, of proefde/rook u niets**?	☐	☐	☐	☐	☐	☐	☐
11	had u last van **oorpijn of oorsuizen**?	☐	☐	☐	☐	☐	☐	☐
12	had u last van een **brok in uw keel of keelpijn**?	☐	☐	☐	☐	☐	☐	☐
13	had u last van **kauw- en/of slikproblemen**?	☐	☐	☐	☐	☐	☐	☐
14	had u last van **zware armen of benen**?	☐	☐	☐	☐	☐	☐	☐
15	had u last van **gewrichts- en/of spierpijn**?	☐	☐	☐	☐	☐	☐	☐
16	had u last van **tintelingen in uw handen, voeten, armen of benen**?	☐	☐	☐	☐	☐	☐	☐
17	had u last van **hoofdpijn**?	☐	☐	☐	☐	☐	☐	☐
18	had u last van **duizeligheid**?	☐	☐	☐	☐	☐	☐	☐
19	had u last van **concentratie- en/of geheugenproblemen**?	☐	☐	☐	☐	☐	☐	☐
20	had u het gevoel **overprikkeld** te zijn?	☐	☐	☐	☐	☐	☐	☐
21	had u last van **nare herinneringen, herbelevingen of nachtmerries**?	☐	☐	☐	☐	☐	☐	☐
22	had u last van **wazig en/of dubbelzien, droge of pijnlijke ogen**?	☐	☐	☐	☐	☐	☐	☐

	In de afgelopen 2 weken, in welke mate …	0<br>Helemaal niet	1<br>Heel weinig	2<br>Een beetje	3<br>Tamelijk	4<br>Erg	5<br>Heel erg	6<br>Volledig
23	had u last van **buikklachten**?	□	□	□	□	□	□	□
24	had u last van u zich **afwisselend warm en koud voelen of had u koorts**?	□	□	□	□	□	□	□
25	had u last van een **droge of pijnlijke huid**?	□	□	□	□	□	□	□
26	had u last van **haaruitval**?	□	□	□	□	□	□	□
	In de afgelopen 2 weken …	Niet	2-3 uur	4-10 uur	11-13 uur	14-24 uur	24-48 uur	langer dan 48 uur
27	als u zich **slechter voelde na het doen van een inspanning**, hoe lang hield dat dan aan?	□	□	□	□	□	□	□
28	had u last van **klachten anders dan die hierboven al genoemd zijn**?	Ik had last van:						

Waarin voelde u zich beperkt?

	In de afgelopen week, in welke mate …	0<br>Helemaal niet	1<br>Heel weinig	2<br>Een beetje	3<br>Tamelijk	4<br>Erg	5<br>Heel erg	6<br>Volledig
29	voelde u zich beperkt in **zware lichamelijke activiteiten** (trap lopen, haasten, sporten)?	□	□	□	□	□	□	□
30	voelde u zich beperkt in **matige lichamelijke activiteiten** (wandelen, huishoudelijk werk, boodschappen doen)?	□	□	□	□	□	□	□
31	voelde u zich beperkt in **dagelijkse activiteiten** (u zelf aankleden, wassen)?	□	□	□	□	□	□	□
32	voelde u zich beperkt in uw **werk** (betaald werk, onbetaald werk, vrijwilligerswerk)?	□	□	□	□	□	□	□
33	voelde u zich beperkt in het u **verplaatsen buitenshuis**?	□	□	□	□	□	□	□
34	voelde u zich beperkt in uw **sociale activiteiten** (uitjes, vrienden en familie bezoeken)?	□	□	□	□	□	□	□
35	had uw aandoening een negatieve invloed op uw **relatie met anderen**?	□	□	□	□	□	□	□
36	had u moeite met **intimiteit** of **seksualiteit**?	□	□	□	□	□	□	□
37	maakte u zich **zorgen over uw toekomst**?	□	□	□	□	□	□	□

Hoe ging het met uw leefstijl?

38	In de afgelopen week, hoeveel dagen heeft u 30 minuten of meer matig intensieve **lichaamsbeweging** gehad?<br>Bijv. stevig doorwandelen of harder fietsen.<br>Het mogen ook minimaal 3x10 minuten zijn.	□ 0 dagen<br>□ 1-2 dagen<br>□ 3-4 dagen<br>□ 5 dagen of meer
39	Hoeveel glazen **alcohol** dronk u in de afgelopen week?	… glazen per week
40	Rookt u of heeft u **gerookt**?	□ Ja. Hoeveel (shag)sigaretten heeft u in de afgelopen week gemiddeld per dag gerookt? ____________<br>□ Vroeger. Gestopt met roken sinds: ___(maand) / ___(jaar)<br>□ Nooit
41	Wat is uw **gewicht**?	……….. kg
42	Wat is uw **lengte**?	……….. cm
43	In de afgelopen week, hoeveel dagen heeft u **gezond gegeten**?	□ 0 dagen<br>□ 1-2 dagen<br>□ 3-4 dagen<br>□ 5 dagen of meer
44	Is er nog iets wat u wilt **bespreken** of waar u **meer informatie** over wilt krijgen?<br>____<br>____	
