# Supplementary material for: Patient perspectives on the usability and content validity of the assessment of burden of chronic conditions tool for post-COVID in the Netherlands: a qualitative study
Source: BMJ Open. 2025 Dec 11;15(12):e109201. doi: 10.1136/bmjopen-2025-109201 (PMC12706107; doi:10.1136/bmjopen-2025-109201)
Supplement: Supplementary data [file bmjopen-15-12-s002.pdf]

APPENDIX 2: TOPIC LIST (ORIGINALLY IN DUTCH)

Demographic questions

1. What is your age?
2. How is your health now?
    a. Do you have other chronic conditions?
3. How was your health before you had post-COVID?
4. What is your educational level?
5. What is your gender?

Acute COVID-19

6. When did you have acute COVID-19?
7. Were you admitted to the hospital?
    a. If yes: where you admitted to the intensive care?
8. How did you get the diagnosis?

Post-COVID

9. Since when do you have post-COVID symptoms?
10. By whom and how where you diagnosed with post-COVID

ABCC-tool

11. How did you discover the ABCC-tool?
12. When and how many times did you complete the ABCC-tool?
13. Would you like to use the ABCC-tool again in the future?
14. Why did you complete the ABCC-tool?
    a. What was your purpose with it?
    b. Did the ABCC-tool meet those purposes?

Content validation

15. Was the ABCC-tool complete (comprehensive)?
16. Was question … relevant for you? (per item in the questionnaire)
17. Was question … formulated clearly? (per item in the questionnaire)
18. What were your thoughts about the answer options?

Usability

19. How was the ABCC-tool online to use?
    a. Was it easy to log in on the app/website?
    b. Was the app/website clear?
20. How much time did it take?
21. What were your thoughts about the balloon diagram?
    a. Was it clear?
    b. Would you like to change anything about it?
22. Did you discuss the balloon diagram with somebody?
    a. If yes: with whom? And why?
23. In what way has the balloon diagram helped you explain how you feel?
    a. Did it give you more compassion from a healthcare professional?
    b. Did it give you more compassion from family or friends?
24. Would you recommend the ABCC-tool to others?
    a. If yes: to whom do you want to recommend the ABCC-tool?
