# Supplementary material for: Patient perspectives on the usability and content validity of the assessment of burden of chronic conditions tool for post-COVID in the Netherlands: a qualitative study
Source: BMJ Open. 2025 Dec 11;15(12):e109201. doi: 10.1136/bmjopen-2025-109201 (PMC12706107; doi:10.1136/bmjopen-2025-109201)
Supplement: Author's manuscript [file bmjopen-2025-109201.draft_revisions.pdf]

BMJ Open is committed to open peer review. As part of this commitment we make the peer review history of every article we publish publicly available.

When an article is published we post the peer reviewers' comments and the authors' responses online. We also post the versions of the paper that were used during peer review. These are the versions that the peer review comments apply to.

The versions of the paper that follow are the versions that were submitted during the peer review process. They are not the versions of record or the final published versions. They should not be cited or distributed as the published version of this manuscript.

BMJ Open is an open access journal and the full, final, typeset and author-corrected version of record of the manuscript is available on our site with no access controls, subscription charges or pay-per-view fees (http://bmjopen.bmj.com).

If you have any questions on BMJ Open's open peer review process please email info.bmjopen@bmj.com

BMJ Open

Patient perspectives on the usability and content validity of the Assessment of Burden of Chronic Conditions (ABCC-) tool for post-COVID: a qualitative study

Journal:	*BMJ Open*
Manuscript ID	bmjopen-2025-109201
Article Type:	Original research
Date Submitted by the Author:	11-Aug-2025
Complete List of Authors:	Debie, Valerie ; Maastricht University, Care and Public Health Research Institute (CAPHRI), Department of Family Medicine<br>Peters, Loraine H L; Maastricht University Care and Public Health Research Institute, Care and Public Health Research Institute (CAPHRI), Department of Family Medicine<br>van Schayck, Onno; Maastricht University, Care and Public Health Research Institute (CAPHRI), Department of Family Medicine<br>Burgers, Jako S.; Maastricht University, Care and Public Health Research Institute (CAPHRI), Department of Family Medicine<br>Ottenheijm, Ramon; Maastricht University, Care and Public Health Research Institute (CAPHRI), Department of Family Medicine<br>Gidding-Slok, Annerika; Maastricht University, Care and Public Health Research Institute (CAPHRI), Department of Family Medicine
Keywords:	Post-Acute COVID-19 Syndrome, Patient Reported Outcome Measures, Chronic Disease

SCHOLARONE™
Manuscripts

I, the Submitting Author has the right to grant and does grant on behalf of all authors of the Work (as defined in the below author licence), an exclusive licence and/or a non-exclusive licence for contributions from authors who are: i) UK Crown employees; ii) where BMJ has agreed a CC-BY licence shall apply, and/or iii) in accordance with the terms applicable for US Federal Government officers or employees acting as part of their official duties; on a worldwide, perpetual, irrevocable, royalty-free basis to BMJ Publishing Group Ltd ("BMJ") its licensees and where the relevant Journal is co-owned by BMJ to the co-owners of the Journal, to publish the Work in this journal and any other BMJ products and to exploit all rights, as set out in our licence.

The Submitting Author accepts and understands that any supply made under these terms is made by BMJ to the Submitting Author unless you are acting as an employee on behalf of your employer or a postgraduate student of an affiliated institution which is paying any applicable article publishing charge ("APC") for Open Access articles. Where the Submitting Author wishes to make the Work available on an Open Access basis (and intends to pay the relevant APC), the terms of reuse of such Open Access shall be governed by a Creative Commons licence – details of these licences and which Creative Commons licence will apply to this Work are set out in our licence referred to above.

Other than as permitted in any relevant BMJ Author's Self Archiving Policies, I confirm this Work has not been accepted for publication elsewhere, is not being considered for publication elsewhere and does not duplicate material already published. I confirm all authors consent to publication of this Work and authorise the granting of this licence.

For peer review only

Patient perspectives on the usability and content validity of the Assessment of Burden of Chronic Conditions (ABCC-) tool for post-COVID: a qualitative study

Short title: ABCC-tool for Post-COVID

For peer review only

V.H.J. Debie[1], L.H.L. Peters[1], O.C.P. van Schayck[1], J.S. Burgers[1], R.P.G. Ottenheijm[1], A.H.M. Gidding-Slok[1]

[1] Department of Family Medicine, Care and Public Health Research Institute (CAPHRI), Maastricht University, Maastricht, the Netherlands.

Corresponding author:

Valerie Debie

P.O Box 616

6200 MD

Maastricht, the Netherlands

v.debie@maastrichtuniversity.nl

For peer review only - http://bmjopen.bmj.com/site/about/guidelines.xhtml

0031 88 388 7060

For peer review only

For peer review only - http://bmjopen.bmj.com/site/about/guidelines.xhtml

Abstract

Background: Post-COVID syndrome manifests with a diverse array of symptoms for which no standard care plan currently exists. Many questions were raised by patients, which underscored the need for a validated patient-reported outcome measure (PROM). Therefore, a post-COVID module was developed to be included in the Assessment of Burden Chronic Conditions tool (ABCC-) tool. The ABCC-tool evaluates and visualizes the perceived physical, emotional, and social burden of one or multiple chronic disease(s) using a balloon diagram, and aims to facilitate person-centered care and structured discussions between patients and healthcare professionals. This study explores the patients' perspective on the content of the ABCC-tool for post-COVID, and the tool's usability.

Methods: All patients who completed the ABCC-tool for post-COVID were invited for an online semi-structured interview. We selected post-COVID patients who had used the tool in the past three months. Interviews were audio recorded and analyzed using a thematic approach with Atlas.ti version 23.

Results: Nineteen post-COVID patients (10 males, mean age 56) were interviewed between May and August 2024. The tool was regarded as user-friendly, and patients indicated they would use the tool again in the future. Patients valued the tool's broad range of topics, some of which are often overlooked in standard healthcare consultations. The tool was

For peer review only - http://bmjopen.bmj.com/site/about/guidelines.xhtml

comprehensible and relevant according to all patients. The balloon diagram was easy to understand, but a legend explaining the colors of the balloons was preferred. Other suggestions for improvement included adding open-text fields, periodic reminders to increase usability, and long-term results.

Conclusion: The ABCC-tool is a promising instrument for post-COVID patients, offering a structured way to monitor and communicate symptoms in addition to standard healthcare consultations. Refinements addressing usability and comprehensiveness are recommended to facilitate its integration into clinical practices.

Keywords

Post-COVID, ABCC-tool, patient reported outcome measure, burden of disease

Strengths and limitations of this study

- The qualitative design provided in depth information about the content and patients' perspective of the ABCC-tool
- Data was analyzed using inductive and deductive approaches to increase the study's credibility

For peer review only

For peer review only - http://bmjopen.bmj.com/site/about/guidelines.xhtml

- Due to the lack of a clinical test for post-COVID, it was not possible to verify the patient's diagnosis

For peer review only - http://bmjopen.bmj.com/site/about/guidelines.xhtml

Introduction

Most people infected with SARS-CoV-2 (COVID-19) fully recover after the acute phase of the disease, regardless of the severity of their infection. However, ten to twenty percent experience persistent symptoms that hinder their daily life activities, and decrease quality of life (QoL) (1, 2). Over 200 different symptoms have been reported, manifesting in various combinations. These may include, but are not limited to, respiratory, cognitive, and neurological symptoms, (chronic) fatigue, and emotional burden (1, 2). This condition, known as post-COVID or long-COVID, is defined by the World Health Organization (WHO) as the emergence and persistence of these symptoms for at least two months, occurring three months after the initial infection, without any other identifiable cause (2).

Post-COVID patients reported persistent symptoms which raise many health-related questions, prompting them to visit their general practitioner (GP). Managing post-COVID is challenging due to the heterogeneity of symptoms and limited knowledge. To address these challenges, it is crucial that GPs take a comprehensive, holistic approach to patient care. This includes managing physical symptoms, supporting mental well-being and social participation, and monitoring recovery. Additionally, GPs could play a role in referring patients to specialists or rehabilitation programs when necessary. However, the absence of evidence-based

For peer review only

For peer review only - http://bmjopen.bmj.com/site/about/guidelines.xhtml

guidelines and the heterogeneous nature of symptoms make this task particularly demanding, and a supporting tool is deemed necessary (3-5).

Given the need for structured support, Lung Foundation Netherlands (an online Dutch forum and helpdesk for lung-related questions) decided in 2020 to develop a COVID-19-oriented tool to help patients with their concerns and coping with their symptoms. Due to the urgent need for patient support, it was decided to use already existing, validated, and well-known patient reported outcome measures (PROMs) as a foundation. Given that acute COVID-19 primarily affects the lungs, PROMs focused on pulmonary symptoms were assessed (6). Eventually, the Assessment of Burden of Chronic obstructive pulmonary disease (ABC-) tool was selected and adjusted to create the Assessment of Burden of COVID-19 (ABCoV-) tool. However, as the pandemic evolved, it became clear that a tool focusing solely on the acute phase of the disease provided only limited value to patients. Therefore, the ABCoV-tool was further adapted to serve as a post-COVID module within the Assessment of Burden of Chronic Conditions (ABCC-) tool.

As the successor to the ABC-tool, the ABCC-tool extends its focus beyond chronic obstructive pulmonary disease (COPD) by including other chronic conditions such as asthma, type 2 diabetes mellitus, chronic heart failure, and osteoarthritis (7-9). The ABCC-tool was chosen because of its person-centered approach and stimulation of self-management and shared

For peer review only

For peer review only - http://bmjopen.bmj.com/site/about/guidelines.xhtml

decision making. These are important factors for disease coping, improving health outcomes, and reducing healthcare services (10). The tool includes a self-administered questionnaire with 41 questions addressing physical, emotional, and social burden of disease, plus one open question, followed by a visual presentation of the results in the form of a balloon diagram (Figure 1) (7, 9). All questions are designed to be understandable at a B1 language level. This level indicates that patients can understand descriptions of events, feelings and wishes, and is characterized with short and easy sentences (11).

The ABCC-tool has been proven to increase perceived quality of care and patient activation (12). A full description of the ABCC-tool and its intended use is published elsewhere (7).

For peer review only

For peer review only - http://bmjopen.bmj.com/site/about/guidelines.xhtml

[INSERT FIGURE 1 HERE]

Figure 1: The balloon diagram of the ABCC-tool for post-COVID. This diagram presents the results of the questionnaire from the ABCC-tool. Red balloons indicate a high burden of disease, while green balloons represent a low burden. Yellow and orange balloons depict intermediate scores, and grey balloons show the scores from the previous completion of the ABCC-tool.

In this study, we aimed to assess the content validity of the ABCC-tool for post-COVID and to evaluate its usability from the patients' perspectives.

Methods

Study design

This qualitative study used an interpretative phenomenological approach to explore the experiences of post-COVID patients with the ABCC-tool in the home setting. This method examines how individuals make sense of their experiences with the tool, and provides a structured framework for research design, data collection, and analysis, enabling a detailed exploration of their perspectives (13, 14). The Consolidated Criteria for Reporting Qualitative Research (COREQ) checklist was used to ensure comprehensive and transparent reporting (15). The full questionnaire is presented in Appendix 1.

For peer review only - http://bmjopen.bmj.com/site/about/guidelines.xhtml

Ethical approval was provided by the Medical Ethics Committee of the Maastricht University Medical Centre (METC 2022-3439). All interviewed patients provided written informed consent before the interview.

Study participants

All patients who had used the ABCC-tool online via CuraVista (www.curavista.health) platform were invited for an online interview via email. CuraVista is an online eHealth platform for managing chronic diseases through monitoring and self-management programs. Patients can use this platform individually or together with their healthcare professional (16). The aim was to interview fifteen to twenty patients until data saturation was achieved. Patients were eligible if they had a self-reported diagnosis of post-COVID according to the WHO-definition, completed the ABCC-tool within the past three months to minimize recall bias, and were proficient in the Dutch language. Before the start of the interviews, patients were specifically asked whether they could remember completing the questionnaire and the balloon diagram, and whether they had been diagnosed with post-COVID. Patients were excluded if they could not remember using the tool or seeing the balloon diagram. Patients were invited in small batches to account for potential non-responses and scheduling conflicts.

For peer review only - http://bmjopen.bmj.com/site/about/guidelines.xhtml

Data collection

Data were collected through single individual semi-structured online interviews in Dutch, conducted between May and August 2024 by one female researcher (VHJD), who was trained in qualitative research. Relevant topics for the interview were outlined in a topic guide developed by the research team, which was pilot tested during the first interview. This topic guide was iteratively adjusted between interviews to address emerging themes to enhance data richness, such as the order of the questions. The final topic list is provided in Appendix 2. The two main topics included the content validity of the ABCC-tool and its usability. Prior to the interviews, patients received a printed copy of the tool's questionnaire and the balloon diagram by mail to facilitate discussion. All interviews were audio-recorded with patients' consent. No field notes were made during the interviews. The interviewer had no relationship with any of the patients.

Data analysis

Audio-recordings were subsequently transcribed using a custom implementation of WhisperX, which provides audio transcription based on Whisper (large-v2 model) and speaker diarization. This transcription process was performed locally on Maastricht University infrastructure/hardware (17). Data analysis was conducted using a thematic approach with Atlas.ti version 23, software for qualitative data analysis. To analyze the transcripts, we used

For peer review only

For peer review only - http://bmjopen.bmj.com/site/about/guidelines.xhtml

152 inductive – the process to identify new themes from textual data – and deductive – the process of applying pre-established codes to the transcripts – coding (18). Related codes were grouped into broader categories, and overarching themes were synthesized to gain a complete understanding of the patients' perspectives. This iterative thematic analysis was reviewed and discussed with all members of the research team until consensus was reached. Initially, two researchers (VHJD and LHLP) independently coded 100% and 50% of all transcripts, respectively, to identify initial codes. The coding was compared, and discrepancies were resolved through discussion, enhancing inter-rater reliability.

Patient and public involvement

No patients or public representatives were involved in any part of this study.

Results

In total, 5615 patients were invited for an interview, of which 27 interviews were started and 19 were completed and included in the analysis. Figure 2 presents the process of selecting and including patients. Ten men and nine women participated with a mean age of 56. Furthermore, approximately half of the patients had comorbidities. Eleven patients had a high educational level. No patients with a low educational level were included. In Table 1 the details per patient are presented. The length of the interviews ranged from 20 to 56 minutes. Data saturation was

For peer review only - http://bmjopen.bmj.com/site/about/guidelines.xhtml

achieved for all topics, except comprehensiveness, after sixteen interviews. In no interview, additional persons were present.

[INSERT FIGURE 2 HERE]

Figure 2: flowchart of the inclusion of patients for an interview

For peer review only

For peer review only - http://bmjopen.bmj.com/site/about/guidelines.xhtml

Table 1: Characteristics of the patients

Patient nr.	Age (years)	Gender	Other chronic conditions	Educational level*
1	55	Female	Apnea<br>Lymphedema<br>Thyroid disorder	Medium
2	58	Female	-	High
3	60	Female	Asthma<br>Eczema	Medium
4	51	Female	Asthma	High
5	60	Male	Musculoskeletal disorder<br>Thyroid disorder	High
6	55	Female	-	Medium
7	57	Female	-	High
8	78	Male	Apnea<br>Cardiovascular disease<br>Rheumatic disease	High
9	52	Male	Asthma	Medium
10	51	Male		Medium
11	43	Male	-	Medium
12	64	Male	-	Medium
13	57	Female	-	High
14	65	Male	COPD**	Medium
15	47	Male	-	High
16	41	Female	-	High
17	51	Male	-	High
18	57	Male	Rheumatic disease	High
19	64	Female	-	High

*Dutch educational levels: *Low* = primary school and preparatory vocational secondary education (VMBO); *Medium* = senior general secondary education (HAVO), university preparatory education (VWO) and secondary vocational education and training (MBO); *High* = higher professional education (HBO) and research-oriented education (WO)(19)

** COPD = chronic obstructive pulmonary disease

For peer review only

For peer review only - http://bmjopen.bmj.com/site/about/guidelines.xhtml

General evaluation of the ABCC-tool

Patients appreciated the tool's questionnaire and balloon diagram for providing a clear overview of both their current and past health status. The diversity of questions was also highly valued, especially those topics that were not typically linked to post-COVID by their healthcare professionals.

Patient 12: "I think it's a good tool. I believe it provides a lot of people with valuable insights into certain complaints, which might be related after all, things you wouldn't always immediately link, but that could indeed be connected in some way."

Several patients found it challenging to complete a questionnaire about their health over the past two weeks. They noted having both good and bad days, which made it difficult to answer the questions consistently. Some patients also struggled to remember their health status over the previous two weeks or from their last use of the tool. To address these challenges, some patients suggested incorporating extra text boxes to allow for personal notes.

Patient 13: "And what might be helpful […] is if there were spaces to write something down about your situation at that moment. […] For example, if I

For peer review only

look at my balloons now, I think, yeah, I have no idea what I was doing then, where I was, or if I was doing different things."

Most patients had used the ABCC-tool multiple times or expressed a willingness to use it more frequently. Nonetheless, many reported forgetting about the tool and suggested receiving periodic reminders, such as monthly or quarterly emails. Others mentioned they would only consider using the tool again if their symptoms changed.

Patient 11: "I think if I received an email saying it's been three months since you filled in the ABCC-tool. Click here to fill it in again, I would be more inclined to do it."

Seven patients required a more detailed explanation of how the tool worked, or what to do with it.

Patient 3: "I see the balloons, but I'm not really sure what I'm supposed to do with them."

Usability of the ABCC-tool

Not all patients could recall exactly where they found the ABCC-tool, but most patients had come across the ABCC-tool during online searches aimed at determining whether their symptoms and disease burden aligned with post-COVID. Others were alerted through

For peer review only - http://bmjopen.bmj.com/site/about/guidelines.xhtml

Facebook support groups, an email from C-support (a foundation of the Dutch ministry of Health, Welfare, and Sport that supports and advises people with post-COVID), or by chance through a Google search for treatments.

Patient 18: "C-support, I believe. I get a lot from them because once you're connected with them and that sort of thing, you receive all kinds of information, so to speak."

Nobody experienced any difficulties with the practical use of the ABCC-tool. The website and login process did not present any problems either.

Patient 6: "No, I found it [the website] quite easy."

The aim of the ABCC-tool was not clear to everyone. Some patients thought it was part of a larger study to find an effective treatment for post-COVID, and that their participation would contribute to the development of new treatments or pharmaceutical interventions. However, most patients understood the aim of the tool.

Patient 13: "I thought it was also for a broader study. I didn't realize it was just for myself and possibly a healthcare provider. I actually didn't know that."

For peer review only - http://bmjopen.bmj.com/site/about/guidelines.xhtml

Most patients expressed a willingness to recommend the tool to other post-COVID patients, although some indicated they did not know any other post-COVID patients. One patient mentioned they would only recommend the tool if additional medical support would follow afterwards. Furthermore, two patients expressed a preference for more medical support after completing the ABCC-tool.

Patient 17: "It needs to be monitored. But if someone is consistently red month after month, then something is really wrong. And at that point, someone should be guided, like, hey, I think you need to be doing something else than what you're doing now. […] Or maybe referred to someone else."

Almost all patients found the questionnaire manageable in terms of length, as it could be paused and resumed at any time. However, one patient mentioned a lack of concentration to complete it and found the questionnaire too long.

Patient 7: "Yes, it was a nice experience. And I definitely liked that it can be saved in between. So, you don't have to complete it all at once. I did it in two parts."

For peer review only

For peer review only - http://bmjopen.bmj.com/site/about/guidelines.xhtml

Content of the ABCC-tool

The questions in the questionnaire were clear to all interviewed patients.

> *Patient 2: "Otherwise, I find it very clear. The language is very clear, and also how you rate [the answer from 0-6] it, so to speak, or the choices you have. Yes, it's just very clear to me."*

Regarding the comprehensiveness of the tool's questionnaire, data saturation was not achieved during the interviews. Four patients suggested including open-text fields throughout the questionnaire, allowing respondents to add personal notes about their current health status. This would facilitate easier comparison of responses across assessments. Another patient proposed incorporating a multiple-choice question to document any changes in areas such as health status, work, family situation, or other relevant personal factors.

Additionally, patients recommended adding questions regarding the type of therapy undergone, general pain experiences, balance difficulties, weight fluctuations due to edema, limitations in daily routines, symptom duration, feelings of being understood by government agencies, and restrictions experienced after intense physical activity.

Several suggestions were made to split questions that contain multiple items into separate questions. For example, two patients recommended breaking down the question on low,

For peer review only - http://bmjopen.bmj.com/site/about/guidelines.xhtml

257 moderate, and vigorous physical activities into smaller steps to enable the possibility of more
258 green balloons.

259 All questions were deemed relevant, except for one regarding sexuality, which was considered
260 inappropriate by some in case of sharing the balloon diagram with their employer. Another
261 patient felt this question was irrelevant for older individuals.

262 *Patient 18: "I can imagine that the topic of sexuality in such a chart... it's*
263 *not very convenient if you want to show it to your boss. That's not*
264 *something they should be involved in."*

265 The answer options were well-received, with most patients finding both the wording and
266 number of response choices suitable.

267 *Patient 11: "I think 0 to 6 is good. Actually, very good."*

268 ***Visual representation***

269 Most patients found the colored balloons easy to understand and fun to use. Furthermore, the
270 balloon diagram made it easier to provide the patient with helpful insights into their health
271 status. However, some patients were confused about whether the height of the balloons (e.g.
272 low balloons indicating low burden) or the colors (e.g. green balloon indicating low burden)
273 indicated the burden of disease or were uncertain about the grey balloons and required further

For peer review only - http://bmjopen.bmj.com/site/about/guidelines.xhtml

clarification. Those who had difficulty understanding the balloons suggested adding a legend or marking ‘high’ and ‘low’ burdens of disease on the vertical axis. Another patient expressed interest in seeing more long-term data, e.g. results from the past six or seven assessments.

> *Patient 12: “At least putting something down like high-low or a lot or not. […] That would have been much clearer, yes.”*

All patients recognized their health status in the balloon representation. While some appreciated the simplicity of the balloons for providing a clear overview of their current situation, others found the abundance of red balloons confronting. Furthermore, patients varied in their level of acceptance regarding their situation, with some demonstrating greater acceptance than others.

> *Patient 2: “It was really confronting for me, but it was clear. You really see it there, that was very helpful for me, to really tell myself […] it’s really real. Yes, that sounds a bit strange, but I’ve always kind of hidden it. Like, it’ll be fine, it’ll get better and just keep going. Yes, when you see it like that, you think, but it really is there.”*

Some patients shared the balloon diagram with healthcare professionals to discuss their current and past health, although not all healthcare professionals expressed interest in the

For peer review only - http://bmjopen.bmj.com/site/about/guidelines.xhtml

balloon diagram. Those who preferred to use the ABCC-tool and balloon diagram together with a healthcare professional would like to use it during visits with GPs, physical therapists, occupational therapists, rehabilitation specialists, or company doctors. While not everyone considered discussing the diagram with a healthcare professional, some patients expressed an intention to do so in the future. Others shared the balloon diagram with family, friends, or colleagues to explain their experiences with post-COVID. However, some patients felt they did not need the diagram, as they were able to explain their condition effectively without it. One patient found the diagram too confronting to share with others.

Patient 1: "No, I never showed the balloons. But I do know where my stumbling blocks are and what I need to work on."

Patient 6: "Yes, with my family members, my children. […] I find it nice to explain to them why I can't do certain things at times. Why I'm occasionally a bit down. […] They also understand it better."

Discussion

This study aimed to assess patients' perspectives on using the ABCC-tool for post-COVID, and the content validity of the questionnaire within the tool. As patients are experiential experts in their disease, in-depth interviews with them provided meaningful insights, emphasizing

For peer review only - http://bmjopen.bmj.com/site/about/guidelines.xhtml

308 essential elements for the tool's use. The ABCC-tool was regarded as user-friendly and
309 provided a comprehensive overview of the post-COVID burden.

310 Findings from this study confirm that the tool includes the most commonly reported post-
311 COVID burdens and offers patients the opportunity to document additional concerns through
312 an open-text feature. Furthermore, patients appreciated the tool for multiple reasons. First, the
313 diversity of topics included in the tool, particularly as many of these are not routinely addressed
314 in their healthcare. Second, patients found the tool's language accessible, because of its short
315 sentences and simple wording (20). Third, the length of the questionnaire was acceptable.
316 Fourth, the visualization gave an insightful overview of disease burden. Finally, the tool was
317 easy to find and use online.

318 Since the emergence of post-COVID, some PROMs have been developed and validated to
319 assess the disease burden, such as the Symptom Burden Questionnaire for Long COVID
320 (SBQ-LB), the Post COVID-19 Condition Stigma Questionnaire (PCCSQ), and the Long
321 COVID Symptoms and Severity Score (LC-SSS scale) (21-24). When comparing these tools
322 to the ABCC-tool, we concluded that the ABCC-tool (42 items) is similar in length compared
323 with the LC-SSS (44 items) and PCCSQ tool (40 items), with all three being scored on a 4 to
324 7 point Likert scale (21-24). However, the ABCC-tool stands out, as none of these other tools
325 specifically focus on the core dimensions of disease burden as defined by the WHO (i.e.,

For peer review only - http://bmjopen.bmj.com/site/about/guidelines.xhtml

326 physical, emotional, and social burden) and incorporate a visual representation of the results.
327 It is important, however, to note that while these tools differ in scope and structure, no formal
328 comparison was conducted regarding user experience or completion time. Therefore, although
329 patients rated the ABCC-tool as user-friendly in this study, no conclusions can yet be drawn
330 about its relative usability or efficiency compared to other available post-COVID PROMs.
331 Future studies could explore these aspects in more depth through head-to-head comparisons.

332 In the current study, patients specifically preferred this visual representation. Although the
333 balloon diagram was not immediately clear to everyone, a brief explanation made it fully
334 understandable. Therefore, the inclusion of a legend for the balloon diagram was considered
335 essential. This is congruent with recommendations that have been made in studies with the
336 ABC-, ABCC-, and Assessment of Burden of ColoRectal Cancer (ABCRC-) tool, to facilitate
337 better understanding of the diagram (10, 25, 26). The balloons gave patients understandable
338 insights into their health status, facilitating structured discussions with healthcare
339 professionals. However, some patients had numerous red balloons which felt confronting and
340 negatively impacted on their motivation, for example, to adopt a healthier lifestyle. It is
341 important to note that many of these patients had comorbidities, making it difficult to determine
342 whether their reported burdens were due to post-COVID or these pre-existing conditions.
343 Therefore, incorporating post-COVID into the ABCC-tool is more beneficial than developing a

For peer review only - http://bmjopen.bmj.com/site/about/guidelines.xhtml

stand-alone tool, as the ABCC-tool includes multiple chronic conditions in a single PROM by integrating the relevant modules for each condition (7-9).

Additionally, other adjustments were recommended to make the tool more suitable for use, these include, amongst others, more open-text fields to accommodate personal notes. Given that memory loss and cognitive impairment are well-documented burdens of post-COVID (2), patients often struggle to recall their experiences from previous weeks. This leads to difficulties in distinguishing day-to-day changes. Therefore, patients preferred the option to make notes to ensure more accurate responses. Furthermore, both patients in this study and those utilizing the ABC-, ABCC-, and ABCRC-tool have suggested the implementation of periodic email reminders to encourage more frequent completion of the questionnaire (10, 25, 26). Research indicates that sending email reminders can significantly improve the completion rate of PROMs (22). Patients indicated that the tool would be more relevant if they had more frequent follow-up results to better monitor their progress.

Only limited evidence is available on optimal treatment strategies for post-COVID, as it is a relatively new disease. Currently, most treatment programs involve wait-and-see approaches, physical exercise, and lifestyle recommendations (27, 28). Ideally, post-COVID care should move towards a more personalized and holistic approach. However, this is challenging due to the lack of robust evidence on what interventions are effective per symptom, the high number

For peer review only - http://bmjopen.bmj.com/site/about/guidelines.xhtml

of reported symptoms, and the substantial variability in burden among individuals (2-4). Given these uncertainties, the ABCC-tool could play a role in addressing this gap.

Strengths and limitations

This study has several strengths and limitations. The qualitative study design provided in depth results in the content validation and the perspective of patient of the ABCC-tool. The use of both inductive and deductive approaches strengthened the study's credibility. The inductive coding process minimized confirmation bias by allowing themes and patterns to naturally emerge from the data, rather than being predefined. Meanwhile, deductive analysis reduced researcher bias by cross-verifying themes with existing frameworks (29-32).

Although data saturation was not fully achieved on comprehensiveness, we believe information power was reached, as sample adequacy in qualitative research depends more on the richness and relevance of the data than on saturation. Factors such as the study's aim and the quality of the collected data play an important role in determining whether the sample holds sufficient information for meaningful analysis (33). Due to the complexity of post-COVID and the aforementioned over 200 symptoms that are associated with this condition (2), it is not feasible to include all symptoms in the questionnaire. We therefore decided that this issue can only be solved by incorporating an open question asking complaints other than those already

For peer review only

mentioned. This way, the most prevalent burdens are included into the tool, with space for additional burdens to report.

We interviewed a roughly equal number of men (n=10) and women (n=9), including patients with and without comorbidities. However, we were not able to take into account educational level when including patients, as this information was unavailable beforehand. Despite the random selection no participants with lower educational levels were included. Regardless, we expect the tool's questions to be accessible and readable for individuals with lower education due to their B1 language level.

Our inclusion criteria were not objectively verifiable. First, we could not confirm whether the patient had post-COVID or not, as there is no standardized or validated diagnostic method for this condition available. Currently, diagnoses are typically made by healthcare professionals or self-reported by patients, based on a set of symptoms. Second, it was impossible to verify the timing of patients' last use of the tool online, as these data were not accessible by the research team. To address these limitations, patients were asked at the start of the interview to specify who provided their post-COVID diagnosis and elaborate when this was self-diagnosed. They were also asked to indicate when they last used the ABCC-tool and whether they could remember this to minimize recall bias. If either of these two criteria was not met, the interview was terminated. For some patients, lack of concentration was a significant burden of

For peer review only

For peer review only - http://bmjopen.bmj.com/site/about/guidelines.xhtml

post-COVID, which impacted their ability to participate in lengthy interviews. One interview had to be cut short on the demand of the patient. Additionally, some patients mentioned at the beginning of the interview not to be able to talk longer than a specific time. In that case, the interviewer started with the most important topics of the study (i.e. usability of the tool and content validation of the questionnaire). For the same reason, transcripts were not returned to the patients because this would be too burdensome.

Implications for future research and practice

Minor adjustments, e.g. open text fields and periodic reminders, need to be made to the tool, based on the findings in this research. After these adjustments, a pilot study should be conducted to evaluate the usability and feasibility of the tool in healthcare settings. This study should include interviews with patients and healthcare professionals to evaluate the barriers and facilitators for using the tool. Additionally, evidence-based treatment recommendations for each domain must be developed and integrated into the tool before clinical implementation. Technical aspects, such as seamless integration into electronic health records, should also be addressed to ensure its practical use in healthcare systems. These steps would enable the ABCC-tool to enhance care for post-COVID patients.

For peer review only - http://bmjopen.bmj.com/site/about/guidelines.xhtml

414 Given the positive results of the ABCC-tool in assessing disease burden in patients with post-COVID, further research is needed to explore its implementation in clinical practice. As post-COVID is a relatively new condition, the first step is to establish guidelines supporting person-centered care and shared decision making. The ABCC-tool could play a key role in supporting this by providing a structured and efficient holistic assessment of patients' burden and needs.

419 *Conclusion*

420 The ABCC-tool is seen as a useful tool to assess experienced burden of disease in patients with post-COVID and to monitor the course of symptoms in time. The most appreciated aspects of the tool are the balloon scheme, which presents the current and past health situation of the patient, and the diverse range of questions in the questionnaire. Furthermore, the content of the questionnaire is praised for being comprehensive, comprehensible, and relevant for post-COVID. Based on the current and previous studies on the ABCC-tool, the tool for post-COVID holds significant potential to enhance person-centered care for post-COVID patients and is suitable for integration into clinical practice.

For peer review only - http://bmjopen.bmj.com/site/about/guidelines.xhtml

Declarations

Funding and competing interests

This study was funded by ZonMw (10430302130003) and Pfizer (75242181). The ZonMw grant was received by Jean Muris, with JSB as project leader. The grant of Pfizer was received by AHMGS. More information is available on https://www.pfizer.nl/. The funders had no role in study design, data collection and analysis, decision to publish, or preparation of the manuscript.

Authors' contributions

VHJD, RPGO, and AHMGS were involved in the study design. VHJD conducted the interviews. VHJD and LHLP performed the coding. All authors reviewed the thematic analysis and reached a consensus. Furthermore, all authors contributed to the writing process, provided feedback, and approved the final version of the manuscript.

Ethics approval and consent to participate

Ethical approval was provided by the Medical Ethics Committee of the Maastricht University Medical Centre (METC 2022-3439). All interviewed patients provided written informed consent before the interview.

For peer review only

For peer review only - http://bmjopen.bmj.com/site/about/guidelines.xhtml

444 ### *Availability of data and material*

445 Data will be stored in a repository called DataHub. The DOI will become available shortly.

446 ### *Acknowledgement*

447 We would like to thank all the patients who showed interest in participating in this study.
448 Furthermore, we would like to thank CuraVista for their contribution in the development and
449 implementation of the ABCC-tool. In addition, this research was made possible, in part, using
450 the Data Science Research Infrastructure (DSRI) hosted at Maastricht University.

For peer review only

References

1. Cha C, Baek G. Symptoms and management of long COVID: A scoping review. Journal of Clinical Nursing. 2024;33(1):11-28.

2. World Health Organization. Post COVID-19 codition (Long COVID) 2022 [Available from: https://www.who.int/europe/news-room/fact-sheets/item/post-covid-19-condition.

3. Bonilla H, Peluso MJ, Rodgers K, Aberg JA, Patterson TF, Tamburro R, et al. Therapeutic trials for long COVID-19: A call to action from the interventions taskforce of the RECOVER initiative. Frontiers in immunology. 2023;14:1129459.

4. Greenhalgh T, Sivan M, Delaney B, Evans R, Milne R. Long covid—an update for primary care. bmj. 2022;378.

5. Platteel TN, Koelmans JC, Cianci D, Broers NJH, de Bont E, Cals JWL, et al. Long-term prognosis of adults with moderately severe SARS-CoV-2 lower respiratory tract infection managed in primary care: Prospective cohort study. Eur J Gen Pract. 2025;31(1):2501306.

6. van Noort EMJ, Claessens D, Moor CC, Berg C, Kasteleyn MJ, In 't Veen J, et al. Online Tool for the Assessment of the Burden of COVID-19 in Patients: Development Study. JMIR Form Res. 2021;5(3):e22603.

7. Boudewijns EA, Claessens D, van Schayck OC, Keijsers LC, Salomé PL, Bilo HJ, et al. ABC-tool reinvented: development of a disease-specific 'Assessment of Burden of Chronic Conditions (ABCC)-tool'for multiple chronic conditions. BMC family practice. 2020;21(1):1-7.

8. Debie VHJ, Boymans TAEJ, Ottenheijm RPG, van Schayck OCP, Gidding-Slok AHM. Expanding the ABCC-tool for Osteoarthritis: Development and Content Validation. Osteoarthritis and Cartilage Open 0. 2024:100488.

9. Slok AHM, Chavannes NH, van der Molen T, Rutten-van Mölken MPMH, Kerstjens HAM, Salomé PL, et al. Development of the Assessment of Burden of COPD tool: an integrated tool to measure the burden of COPD. NPJ Primary Care Respiratory Medicine. 2014;24(1):1-4.

10. Slok AH, Twellaar M, Jutbo L, Kotz D, Chavannes NH, Holverda S, et al. 'To use or not to use': a qualitative study to evaluate experiences of healthcare providers and patients with the assessment of burden of COPD (ABC) tool. npj Primary Care Respiratory Medicine. 2016;26(1):1-8.

11. Europass. Het gemeenschappelijk Europees referentiekader voor talen n.d. [Available from: https://europass.europa.eu/nl/common-european-framework-reference-language-skills.

12. Boudewijns EA, Claessens D, van Schayck OC, Twellaar M, Winkens B, Joore MA, et al. Effectiveness of the Assessment of Burden of Chronic Conditions (ABCC)-tool in patients

For peer review only

For peer review only - http://bmjopen.bmj.com/site/about/guidelines.xhtml

with asthma, COPD, type 2 diabetes mellitus, and heart failure: A pragmatic clustered quasi-experimental study in the Netherlands. European Journal of General Practice. 2024;30(1):2343364.

13. Tindall L. J.A. Smith, P. Flower and M. Larkin (2009), Interpretative Phenomenological Analysis: Theory, Method and Research. Qualitative Research in Psychology. 2009;6(4):346-7.

14. Pietkiewicz I, Smith JA. A practical guide to using interpretative phenomenological analysis in qualitative research psychology. Psychological journal. 2014;20(1):7-14.

15. Tong A, Sainsbury P, Craig J. Consolidated criteria for reporting qualitative research (COREQ): a 32-item checklist for interviews and focus groups. Int J Qual Health Care. 2007;19(6):349-57.

16. van Zelst CM, Kasteleyn MJ, van Noort EMJ, Rutten-van Molken M, Braunstahl GJ, Chavannes NH, et al. The impact of the involvement of a healthcare professional on the usage of an eHealth platform: a retrospective observational COPD study. Respir Res. 2021;22(1):88.

17. Radford A, Kim JW, Xu T, Brockman G, McLeavey C, Sutskever I, editors. Robust speech recognition via large-scale weak supervision. International conference on machine learning; 2023: PMLR.

18. Chandra Y, Shang L. Inductive Coding. In: Chandra Y, Shang L, editors. Qualitative Research Using R: A Systematic Approach. Singapore: Springer Nature Singapore; 2019. p. 91-106.

19. CBS. Opleidingsniveau [Available from: https://www.cbs.nl/nl-nl/nieuws/2019/33/verschil-levensverwachting-hoog-en-laagopgeleid-groeit/opleidingsniveau.

20. Ministerie van Algemene Zaken. Taalniveau B1 [Available from: https://www.communicatierijk.nl/vakkennis/rijkswebsites/aanbevolen-richtlijnen/taalniveau-b1.

21. Hughes SE, Haroon S, Subramanian A, McMullan C, Aiyegbusi OL, Turner GM, et al. Development and validation of the symptom burden questionnaire for long covid (SBQ-LC): Rasch analysis. Bmj. 2022;377:e070230.

22. Damant RW, Rourke L, Cui Y, Lam GY, Smith MP, Fuhr DP, et al. Reliability and validity of the post COVID-19 condition stigma questionnaire: A prospective cohort study. EClinicalMedicine. 2023;55:101755.

23. Ye G, Zhu Y, Bao W, Zhou H, Lai J, Zhang Y, et al. The Long COVID Symptoms and Severity Score: Development, Validation, and Application. Value Health. 2024;27(8):1085-91.

24. Tran VT, Riveros C, Clepier B, Desvarieux M, Collet C, Yordanov Y, et al. Development and Validation of the Long Coronavirus Disease (COVID) Symptom and Impact Tools: A Set of Patient-Reported Instruments Constructed From Patients' Lived Experience. Clin Infect Dis. 2022;74(2):278-87.

For peer review only

For peer review only - http://bmjopen.bmj.com/site/about/guidelines.xhtml

25. Thomassen BJ, Gielen AH, Baak J, Sosef M, Ancion E, Gielen CM, et al. Supporting follow-up care in colorectal cancer patients: first experiences with the Assessment of Burden of ColoRectal Cancer (ABCRC)-tool. Journal of Cancer Survivorship. 2024:1-11.

26. Peters LHL, Keijsers LCEM, van Schayck OCP, Muris JWM, Vervloet M, Gidding-Slok AHM. Patient Experiences with the Assessment of Burden of Chronic Conditions (ABCC) Tool in Primary Care: A Qualitative Study. Submitted.

27. Bachmeier BE, Hölzle S, Gasser M, van den Akker M. How do German general practitioners manage long-/post-COVID? A qualitative study in primary care. Viruses. 2023;15(4):1016.

28. Kogel A, Machatschek M, Scharschmidt R, Wollny C, Lordick F, Ghanem M, et al. Physical exercise as a treatment for persisting symptoms post-COVID infection: Review of ongoing studies and prospective randomized controlled training study. Clinical Research in Cardiology. 2023;112(11):1699-709.

29. Fereday J, Muir-Cochrane E. Demonstrating rigor using thematic analysis: A hybrid approach of inductive and deductive coding and theme development. International journal of qualitative methods. 2006;5(1):80-92.

30. Hsieh H-F, Shannon SE. Three approaches to qualitative content analysis. Qualitative health research. 2005;15(9):1277-88.

31. Nickerson RS. Confirmation bias: A ubiquitous phenomenon in many guises. Review of general psychology. 1998;2(2):175-220.

32. Nowell LS, Norris JM, White DE, Moules NJ. Thematic analysis: Striving to meet the trustworthiness criteria. International journal of qualitative methods. 2017;16(1):1609406917733847.

33. Malterud K, Siersma VD, Guassora AD. Sample size in qualitative interview studies: guided by information power. Qualitative health research. 2016;26(13):1753-60.

For peer review only

For peer review only - http://bmjopen.bmj.com/site/about/guidelines.xhtml

Appendix 1: Questionnaire in the ABCC-tool

ENGLISCH VERSION

Dear Sir/Madam,
With this questionnaire, we would like to get an impression of how you are doing. During consultation with your healthcare provider, you can talk about the topics that are important to you.
These questions are related to the chronic condition(s) for which you are visiting the healthcare provider.

What were you suffering from?

	In the past week, how often ….	0 Never	1 Hardly ever	2 A few times	3 Several times	4 Many times	5 A great many times	6 Almost all the time
1	did you suffer from **fatigue**?	☐	☐	☐	☐	☐	☐	☐
2	did you have a poor **night's rest**?	☐	☐	☐	☐	☐	☐	☐
3	did you suffer from sadness, fear, frustration, shame or other **unpleasant feelings**?	☐	☐	☐	☐	☐	☐	☐
4	did you suffer **adjusting your life** (e.g. planning of activities, get enough exercise, eating healthy)?	☐	☐	☐	☐	☐	☐	☐
	In the past 2 weeks, to what extend …	0 Not at all	1 Very slightly	2 Slightly	3 Moderate	4 Very	5 Extremely	6 Totally
5	did you suffer from a **decreased condition**?	☐	☐	☐	☐	☐	☐	☐
6	did you suffer from **pain** or **difficulties with breathing**?	☐	☐	☐	☐	☐	☐	☐
7	did you suffer from **coughing**?	☐	☐	☐	☐	☐	☐	☐
8	did you suffer from **pain on the chest** or **a feeling of pressure on the chest**?	☐	☐	☐	☐	☐	☐	☐
9	did you suffer from **palpitations**?	☐	☐	☐	☐	☐	☐	☐
10	did you suffer from a **strange taste or smell, or tasted/smelled nothing**?	☐	☐	☐	☐	☐	☐	☐

For peer review only - http://bmjopen.bmj.com/site/about/guidelines.xhtml

11	did you suffer from **earache** or **tinnitus**?	☐	☐	☐	☐	☐	☐	☐
12	did you suffer from a **lump in your throat** or a **sore throat**?	☐	☐	☐	☐	☐	☐	☐
13	did you suffer from problems with **chewing and/or swallowing**?	☐	☐	☐	☐	☐	☐	☐
14	did you suffer from **heavy arms or legs**?	☐	☐	☐	☐	☐	☐	☐
15	did you suffer from **joint and/or muscle pain**?	☐	☐	☐	☐	☐	☐	☐
16	did you suffer from **tingling in your hands, feet, arms or legs**?	☐	☐	☐	☐	☐	☐	☐
17	did you suffer from **a headache**?	☐	☐	☐	☐	☐	☐	☐
18	did you suffer from **dizziness**?	☐	☐	☐	☐	☐	☐	☐
19	did you suffer from problems with **concentration and/or memory**?	☐	☐	☐	☐	☐	☐	☐

	In the past 2 weeks, to what extend …	0 Not at all	1 Very slightly	2 Slightly	3 Moderate	4 Very	5 Extremely	6 Totally
20	did you feel **overstimulated**?	☐	☐	☐	☐	☐	☐	☐
21	did you suffer from **bad memories, flashbacks or nightmares**?	☐	☐	☐	☐	☐	☐	☐
22	did you suffer from **blurred and/or double vision, dry or painful eyes**?	☐	☐	☐	☐	☐	☐	☐
23	did you suffer from **abdominal complaints**?	☐	☐	☐	☐	☐	☐	☐
24	did you suffer from **feeling hot and cold alternately** or did you have a **fever**?	☐	☐	☐	☐	☐	☐	☐
25	did you suffer from **dry or sore skin**?	☐	☐	☐	☐	☐	☐	☐
26	did you suffer from **hair loss**?	☐	☐	☐	☐	☐	☐	☐

	In the past 2 weeks, …	Not	2-3 hours	4-10 hours	11-13 hours	14-24 hours	24-48 hours	Longer than 48 hours
27	if you **felt worse after exercising**, how long did this last?	☐	☐	☐	☐	☐	☐	☐

For peer review only - http://bmjopen.bmj.com/site/about/guidelines.xhtml

28	did you suffer from **any complaints other than those mentioned above**?	I suffered from:

In what did you feel limited?

	In the past week, to what extend …	0 Not at all	1 Very slightly	2 Slightly	3 Moderate	4 Very	5 Extremel y	6 Totally
29	were you limited in **strenuous physical** activities (such as climbing stairs, hurrying, doing sports)?	□	□	□	□	□	□	□
30	were you limited in **moderate physical** activities (such as walking, housework, carrying things)?	□	□	□	□	□	□	□
31	were you limited in **daily activities** at home (such as dressing, washing yourself)	□	□	□	□	□	□	□
32	were you limited in your **work or social activities** (short trip, visiting friends and family)?	□	□	□	□	□	□	□
33	had your condition a negative impact on your **relations with others**?	□	□	□	□	□	□	□
34	did you have any difficulty with **intimacy or sexuality**?	□	□	□	□	□	□	□
35	did you **worry about your future**?	□	□	□	□	□	□	□

How was your lifestyle?

36	In the past week, how many days have you had moderately intense **physical exercise** for 30 minutes or more? E.g. walking or cycling in a fast pace	□ 0 days<br>□ 1-2 days<br>□ 3-4 days<br>□ 5 days or more

For peer review only - http://bmjopen.bmj.com/site/about/guidelines.xhtml

	It may also be a minimum of 3x10 minutes	
37	How many glasses of **alcohol** did you drink in the past week?	………… glasses per week
38	Do you **smoke** or have you smoked?	□ Yes. In the last week, how many (shag) cigarettes have you smoked on average per day? ___________<br>□ Previously. Stopped smoking since: ____ (month) / ____ (year)<br>□ Never
39	What is your **weight**?	………… kg
40	What is your **height**?	………… cm
41	In the past week, how many days did you **eat healthy**?	□ 0 days<br>□ 1-2 days<br>□ 3-4 days<br>□ 5 days or more
42	Is there anything else you would like to **discuss** or would like to receive more **information** about? ____________________<br>____________________	

549

For peer review only - http://bmjopen.bmj.com/site/about/guidelines.xhtml

DUTCH VERSION

Beste meneer/mevrouw,
Met deze vragenlijst willen we samen met u in kaart brengen hoe het met u gaat.
Deze vragen hebben te maken met de chronische aandoening(en) waarvoor u bij de zorgverlener komt.

Waar had u last van?

	In de afgelopen week, hoe vaak …	0<br>Nooit	1<br>Zelden	2<br>Af en toe	3<br>Regel-matig	4<br>Heel vaak	5<br>Meestal	6<br>Altijd
1	had u last van **vermoeidheid**?	□	□	□	□	□	□	□
2	had u een slechte **nachtrust**?	□	□	□	□	□	□	□
3	had u last van somberheid, angst, frustratie, schaamte of andere **vervelende gevoelens**?	□	□	□	□	□	□	□
4	had u **moeite om uw leven aan te passen** (bijv. plannen van activiteiten, voldoende beweging, gezond eten)?	□	□	□	□	□	□	□

	In de afgelopen 2 weken, in welke mate …	0<br>Hele-maal niet	1<br>Heel weinig	2<br>Een beetje	3<br>Tamelijk	4<br>Erg	5<br>Heel erg	6<br>Volledig
5	had u last van een **verminderde conditie**?	□	□	□	□	□	□	□
6	had u last van **pijn of moeite met ademhalen**?	□	□	□	□	□	□	□
7	had u last van **hoesten**?	□	□	□	□	□	□	□
8	had u last van **pijn op de borst of een drukkend gevoel op de borst**?	□	□	□	□	□	□	□
9	had u last van **hartkloppingen**?	□	□	□	□	□	□	□
10	had u last van een **vreemde smaak of geur, of proefde/rook u niets**?	□	□	□	□	□	□	□
11	had u last van **oorpijn of oorsuizen**?	□	□	□	□	□	□	□
12	had u last van een **brok in uw keel of keelpijn**?	□	□	□	□	□	□	□
13	had u last van **kauw- en/of slikproblemen**?	□	□	□	□	□	□	□

For peer review only - http://bmjopen.bmj.com/site/about/guidelines.xhtml

14	had u last van **zware armen of benen**?	□	□	□	□	□	□	□
15	had u last van **gewrichts- en/of spierpijn**?	□	□	□	□	□	□	□
16	had u last van **tintelingen in uw handen, voeten, armen of benen**?	□	□	□	□	□	□	□
17	had u last van **hoofdpijn**?	□	□	□	□	□	□	□
18	had u last van **duizeligheid**?	□	□	□	□	□	□	□
19	had u last van **concentratie- en/of geheugenproblemen**?	□	□	□	□	□	□	□
20	had u het gevoel **overprikkeld** te zijn?	□	□	□	□	□	□	□
21	had u last van **nare herinneringen, herbelevingen of nachtmerries**?	□	□	□	□	□	□	□
22	had u last van **wazig en/of dubbelzien, droge of pijnlijke ogen**?	□	□	□	□	□	□	□
	In de afgelopen 2 weken, in welke mate …	0<br>Hele-maal niet	1<br>Heel weinig	2<br>Een beetje	3<br>Tamelijk	4<br>Erg	5<br>Heel erg	6<br>Volledig
23	had u last van **buikklachten**?	□	□	□	□	□	□	□
24	had u last van u zich **afwisselend warm en koud voelen of had u koorts**?	□	□	□	□	□	□	□
25	had u last van een **droge of pijnlijke huid**?	□	□	□	□	□	□	□
26	had u last van **haaruitval**?	□	□	□	□	□	□	□
	In de afgelopen 2 weken …	Niet	2-3 uur	4-10 uur	11-13 uur	14-24 uur	24-48 uur	langer dan 48 uur
27	als u zich **slechter voelde na het doen van een inspanning**, hoe lang hield dat dan aan?	□	□	□	□	□	□	□
28	had u last van **klachten anders dan die hierboven al genoemd zijn**?	Ik had last van:						

Waarin voelde u zich beperkt?

	In de afgelopen week, in welke mate …	0<br>Hele-maal niet	1<br>Heel weinig	2<br>Een beetje	3<br>Tamelijk	4<br>Erg	5<br>Heel erg	6<br>Volledig

For peer review only - http://bmjopen.bmj.com/site/about/guidelines.xhtml

29	voelde u zich beperkt in **zware lichamelijke activiteiten** (trap lopen, haasten, sporten)?	□	□	□	□	□	□	□
30	voelde u zich beperkt in **matige lichamelijke activiteiten** (wandelen, huishoudelijk werk, boodschappen doen)?	□	□	□	□	□	□	□
31	voelde u zich beperkt in **dagelijkse activiteiten** (u zelf aankleden, wassen)?	□	□	□	□	□	□	□
32	voelde u zich beperkt in uw **werk of sociale activiteiten** (uitjes, vrienden en familie bezoeken)?	□	□	□	□	□	□	□
33	had uw aandoening een negatieve invloed op uw **relatie met anderen**?	□	□	□	□	□	□	□
34	had u moeite met **intimiteit** of **seksualiteit**?	□	□	□	□	□	□	□
35	maakte u zich **zorgen over uw toekomst**?	□	□	□	□	□	□	□

Hoe ging het met uw leefstijl?

36	In de afgelopen week, hoeveel dagen heeft u 30 minuten of meer matig intensieve **lichaamsbeweging** gehad? Bijv. stevig doorwandelen of harder fietsen.	□ 0 dagen<br>□ 1-2 dagen<br>□ 3-4 dagen<br>□ 5 dagen of meer

For peer review only - http://bmjopen.bmj.com/site/about/guidelines.xhtml

	Het mogen ook minimaal 3x10 minuten zijn.	
37	Hoeveel glazen **alcohol** dronk u in de afgelopen week?	… glazen per week
38	Rookt u of heeft u **gerookt**?	□ Ja. Hoeveel (shag)sigaretten heeft u in de afgelopen week gemiddeld per dag gerookt? ___________<br>□ Vroeger. Gestopt met roken sinds: ___(maand) / ___(jaar)<br>□ Nooit
39	Wat is uw **gewicht**?	………… kg
40	Wat is uw **lengte**?	………… cm
41	In de afgelopen week, hoeveel dagen heeft u **gezond gegeten**?	□ 0 dagen<br>□ 1-2 dagen<br>□ 3-4 dagen<br>□ 5 dagen of meer
42	Is er nog iets wat u wilt **bespreken** of waar u **meer informatie** over wilt krijgen?<br>____<br>____________________________________	

For peer review only

For peer review only - http://bmjopen.bmj.com/site/about/guidelines.xhtml

Appendix 2: Topic list (originally in Dutch)

Demographic questions

1. What is your age?
2. How is your health now?
3. How was your health before you had post-COVID?
4. What is your educational level?
5. What is your gender?

Acute COVID-19

6. When did you have acute COVID-19?
7. Were you admitted to the hospital?
    a. If yes: where you admitted to the intensive care?
8. How did you get the diagnosis?

Post-COVID

9. Since when do you have post-COVID symptoms?
10. Who and how where you diagnosed with post-COVID

ABCC-tool

11. How did you discover the ABCC-tool?
12. When and how many times did you complete the ABCC-tool?
13. Would you like to use the ABCC-tool again in the future?
14. Why did you complete the ABCC-tool?
    a. What was your purpose with it?
    b. Did the ABCC-tool meet those purposes?

Content validation

15. Was the ABCC-tool complete?
16. Were all the questions relevant for you?
17. Were all questions formulated clearly?
18. What were your thoughts about the answer options?

Usability

19. How was the ABCC-tool online to use?
    a. Was it easy to log in on the app/website?
    b. Was the app/website clear?

For peer review only

20. How much time did it take?
21. What were your thoughts about the balloon diagram?
    a. Was it clear?
    b. Would you like to change anything about it?
22. Did you discuss the balloon diagram with somebody?
    a. If yes: with whom? And why?
23. In what way has the balloon diagram helped you explain how you feel?
    a. Did it give you more compassion from a healthcare professional?
    b. Did it give you more compassion from family or friends?
24. Would you recommend the ABCC-tool to others?
    a. If yes: to whom do you want to recommend the ABCC-tool?

For peer review only

For peer review only - http://bmjopen.bmj.com/site/about/guidelines.xhtml

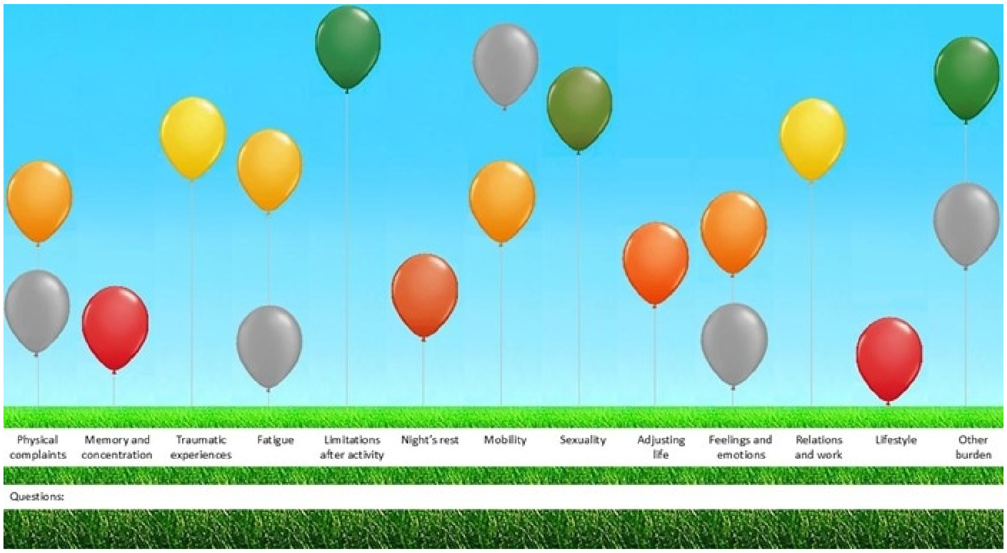

Figure 1: The balloon diagram of the ABCC-tool for post-COVID. This diagram presents the results of the questionnaire from the ABCC-tool. Red balloons indicate a high burden of disease, while green balloons represent a low burden. Yellow and orange balloons depict intermediate scores, and grey balloons show the scores from the previous completion of the ABCC-tool.

185x101mm (96 x 96 DPI)

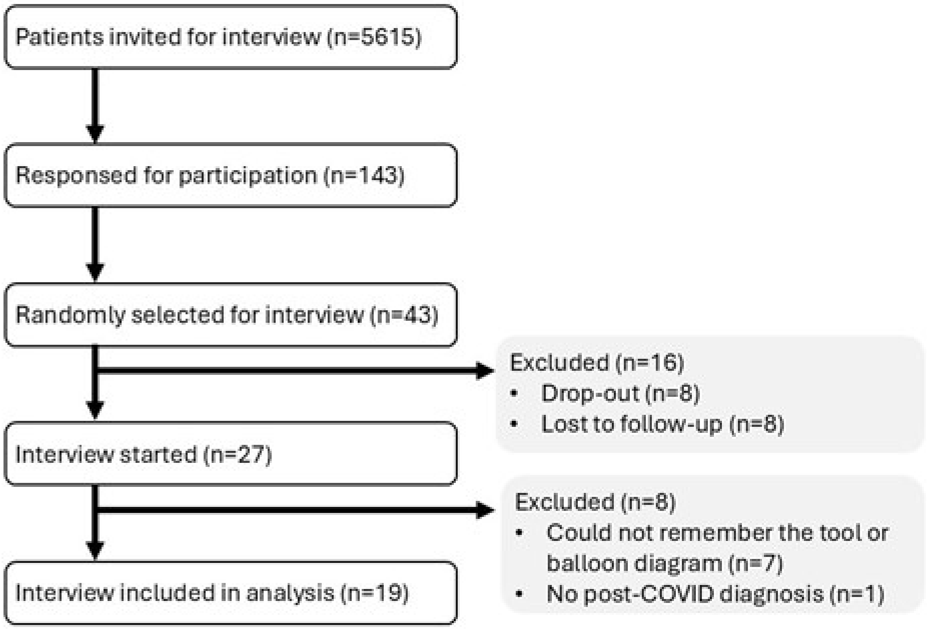

Figure 2: flowchart of the inclusion of patients for an interview

127x85mm (96 x 96 DPI)

BMJ Open

Patient perspectives on the usability and content validity of the Assessment of Burden of Chronic Conditions (ABCC-) tool for post-COVID: a qualitative study

Journal:	*BMJ Open*
Manuscript ID	bmjopen-2025-109201.R1
Article Type:	Original research
Date Submitted by the Author:	21-Oct-2025
Complete List of Authors:	Debie, Valerie ; Maastricht University, Care and Public Health Research Institute (CAPHRI), Department of Family Medicine<br>Peters, Loraine H L; Maastricht University Care and Public Health Research Institute, Care and Public Health Research Institute (CAPHRI), Department of Family Medicine<br>van Schayck, Onno; Maastricht University, Care and Public Health Research Institute (CAPHRI), Department of Family Medicine<br>Burgers, Jako S.; Maastricht University, Care and Public Health Research Institute (CAPHRI), Department of Family Medicine<br>Ottenheijm, Ramon; Maastricht University, Care and Public Health Research Institute (CAPHRI), Department of Family Medicine<br>Gidding-Slok, Annerika; Maastricht University, Care and Public Health Research Institute (CAPHRI), Department of Family Medicine
**Primary Subject Heading**:	Patient-centred medicine
Secondary Subject Heading:	Qualitative research
Keywords:	Post-Acute COVID-19 Syndrome, Patient Reported Outcome Measures, Chronic Disease

SCHOLARONE™
Manuscripts

I, the Submitting Author has the right to grant and does grant on behalf of all authors of the Work (as defined in the below author licence), an exclusive licence and/or a non-exclusive licence for contributions from authors who are: i) UK Crown employees; ii) where BMJ has agreed a CC-BY licence shall apply, and/or iii) in accordance with the terms applicable for US Federal Government officers or employees acting as part of their official duties; on a worldwide, perpetual, irrevocable, royalty-free basis to BMJ Publishing Group Ltd ("BMJ") its licensees and where the relevant Journal is co-owned by BMJ to the co-owners of the Journal, to publish the Work in this journal and any other BMJ products and to exploit all rights, as set out in our licence.

The Submitting Author accepts and understands that any supply made under these terms is made by BMJ to the Submitting Author unless you are acting as an employee on behalf of your employer or a postgraduate student of an affiliated institution which is paying any applicable article publishing charge ("APC") for Open Access articles. Where the Submitting Author wishes to make the Work available on an Open Access basis (and intends to pay the relevant APC), the terms of reuse of such Open Access shall be governed by a Creative Commons licence – details of these licences and which Creative Commons licence will apply to this Work are set out in our licence referred to above.

Other than as permitted in any relevant BMJ Author's Self Archiving Policies, I confirm this Work has not been accepted for publication elsewhere, is not being considered for publication elsewhere and does not duplicate material already published. I confirm all authors consent to publication of this Work and authorise the granting of this licence.

For peer review only

Patient perspectives on the usability and content validity of the Assessment of Burden of Chronic Conditions (ABCC-) tool for post-COVID: a qualitative study

Short title: ABCC-tool for Post-COVID

For peer review only

V.H.J. Debie[1], L.H.L. Peters[1], O.C.P. van Schayck[1], J.S. Burgers[1], R.P.G. Ottenheijm[1], A.H.M. Gidding-Slok[1]

[1] Department of Family Medicine, Care and Public Health Research Institute (CAPHRI), Maastricht University, Maastricht, the Netherlands.

Corresponding author:

Valerie Debie

P.O Box 616

6200 MD

Maastricht, the Netherlands

v.debie@maastrichtuniversity.nl

For peer review only - http://bmjopen.bmj.com/site/about/guidelines.xhtml

0031 88 388 7060

For peer review only

For peer review only - http://bmjopen.bmj.com/site/about/guidelines.xhtml

Abstract

Background: Post-COVID syndrome manifests with a diverse array of symptoms for which no standard care plan currently exists. Many questions were raised by patients, which underscored the need for a validated patient-reported outcome measure (PROM). Therefore, a post-COVID module was developed to be included in the Assessment of Burden Chronic Conditions tool (ABCC-) tool. The ABCC-tool evaluates and visualizes the perceived physical, emotional, and social burden of one or multiple chronic disease(s) using a balloon diagram, and aims to facilitate person-centered care and structured discussions between patients and healthcare professionals. This study explores the patients' perspective on the content of the ABCC-tool for post-COVID, and the tool's usability in a home-based setting.

Methods: All patients who completed the ABCC-tool for post-COVID were invited for an online semi-structured interview. We selected post-COVID patients who had used the tool in the past three months. Interviews were audio recorded and analyzed using a thematic approach with Atlas.ti version 23.

Results: Nineteen post-COVID patients (10 males, mean age 56) were interviewed between May and August 2024. The tool was regarded as user-friendly, and patients indicated they would use the tool again in the future. Patients valued the tool's broad range of topics, some of which are often overlooked in standard healthcare consultations. The tool was

For peer review only

For peer review only - http://bmjopen.bmj.com/site/about/guidelines.xhtml

comprehensible and relevant according to all patients. The balloon diagram was easy to understand, but a legend explaining the colors of the balloons was preferred. Other suggestions for improvement included adding open-text fields, periodic reminders to increase usability, and long-term results.

Conclusion: The ABCC-tool is a promising instrument for post-COVID patients, offering a structured way to monitor and communicate experienced burden in addition to standard healthcare consultations. Refinements addressing usability and comprehensiveness are recommended to facilitate its integration into clinical practices.

Keywords

Post-COVID, ABCC-tool, patient reported outcome measure, burden of disease

Strengths and limitations of this study

- The qualitative design provided in depth information about the content and patients' perspective of the ABCC-tool
- Data was analyzed using inductive and deductive approaches to increase the study's credibility

For peer review only

- Due to the lack of a clinical test for post-COVID, it was not possible to verify the patient's diagnosis

For peer review only - http://bmjopen.bmj.com/site/about/guidelines.xhtml

Introduction

Most people infected with SARS-CoV-2 (COVID-19) fully recover after the acute phase of the disease, regardless of the severity of their infection. However, according to the World Health Organization (WHO), six percent experience persistent symptoms that hinder their daily life activities, and decrease quality of life (QoL) [1-3]. Over 200 different symptoms have been reported, manifesting in various combinations. These may include, but are not limited to, respiratory, cognitive, and neurological symptoms, (chronic) fatigue, emotional burden, and work impairment [1,2,4]. This condition, known as post-COVID or long-COVID, is defined by the WHO as the emergence and persistence of these symptoms for at least two months, occurring three months after the initial infection, without any other identifiable cause [2].

Post-COVID patients reported persistent symptoms which raise many health-related questions, prompting them to visit their general practitioner (GP). Managing post-COVID is challenging due to the heterogeneity of symptoms and limited knowledge. To address these challenges, it is crucial that GPs take a comprehensive, holistic approach to patient care. This includes managing physical symptoms, supporting mental well-being and social participation, and monitoring recovery. Additionally, GPs could play a role in referring patients to specialists or rehabilitation programs when necessary. However, the absence of evidence-based

For peer review only

For peer review only - http://bmjopen.bmj.com/site/about/guidelines.xhtml

guidelines and the heterogeneous nature of symptoms make this task particularly demanding, and a supporting tool is deemed necessary [5-7].

Given the need for structured support, Lung Foundation Netherlands (an online Dutch forum and helpdesk for lung-related questions) decided in 2020 to develop a COVID-19-oriented tool to help patients with their concerns and coping with their symptoms. Due to the urgent need for patient support, it was decided to use already existing, validated, and well-known patient reported outcome measures (PROMs) as a foundation. Given that acute COVID-19 primarily affects the lungs, PROMs focused on pulmonary symptoms were assessed [8]. Eventually, the Assessment of Burden of Chronic obstructive pulmonary disease (ABC-) tool was selected and adjusted to create the Assessment of Burden of COVID-19 (ABCoV-) tool [8]. However, as the pandemic evolved, it became clear that a tool focusing solely on the acute phase of the disease provided only limited value to patients. Therefore, the ABCoV-tool was further adapted to serve as a post-COVID module within the Assessment of Burden of Chronic Conditions (ABCC-) tool.

As the successor to the ABC-tool, the ABCC-tool extends its focus beyond chronic obstructive pulmonary disease (COPD) by including other chronic conditions such as asthma, type 2 diabetes mellitus, chronic heart failure, and osteoarthritis [9-11]. The ABCC-tool was chosen because it measures burden of disease on a physical, emotional and social level, and because

For peer review only - http://bmjopen.bmj.com/site/about/guidelines.xhtml

of its person-centered approach and stimulation of self-management and shared decision making. These are important factors for disease coping, improving health outcomes, and reducing the use of healthcare services [12]. The tool includes a self-administered questionnaire with 41 questions addressing physical, emotional, and social burden of disease, plus one open question, followed by a visual presentation of the results in the form of a balloon diagram (Figure 1) [9,11]. All questions are designed to be understandable at a B1 language level. This level indicates that patients can understand descriptions of events, feelings and wishes, and is characterized with short and easy sentences [13].

The ABCC-tool has been proven to increase perceived quality of care and patient activation [14]. A full description of the ABCC-tool and its intended use is published elsewhere [9].

For peer review only

For peer review only - http://bmjopen.bmj.com/site/about/guidelines.xhtml

[INSERT FIGURE 1 HERE]

Figure 1: The balloon diagram of the ABCC-tool for post-COVID. This diagram presents the results of the questionnaire from the ABCC-tool. Red balloons indicate a high burden of disease, while green balloons represent a low burden. Yellow and orange balloons depict intermediate scores, and grey balloons show the scores from the previous completion of the ABCC-tool.

In this study, we aimed to assess the content validity of the ABCC-tool for post-COVID and to evaluate its usability from the patients' perspectives. To the best of our knowledge, no other tool has the capability to measure physical, emotional, and social disease burden of post-COVID with the possibility to measure these disease burden in other chronic conditions, and visualize the results. Such tools are highly demanded by patients. Therefore, this evaluation was to ensure that the tool adequately covers all relevant content and to identify potential areas for improvement to make it easier for patients to use.

For peer review only

Methods

Study design

This qualitative study used an interpretative phenomenological approach to explore the experiences of post-COVID patients with the ABCC-tool in the home setting. This method

For peer review only - http://bmjopen.bmj.com/site/about/guidelines.xhtml

examines how individuals make sense of their experiences with the tool, and provides a structured framework for research design, data collection, and analysis, enabling a detailed exploration of their perspectives [15,16]. The COSMIN methodology for assessing the content validity of PROMs was used to assess the content validity [17]. The Consolidated Criteria for Reporting Qualitative Research (COREQ) checklist was used to ensure comprehensive and transparent reporting [18]. The full questionnaire is presented in Appendix 1.

Ethical approval was provided by the Medical Ethics Committee of the Maastricht University Medical Centre (METC 2022-3439). The study was funded by Pfizer and ZonMw, however, they did not have any involvement in the study design, data collection, analysis or manuscript writing process. All interviewed patients provided written informed consent before the interview.

Study participants

All patients who had used the ABCC-tool online via CuraVista (www.curavista.health) platform were invited for an online interview via email by CuraVista. CuraVista is an online eHealth platform for managing chronic diseases through monitoring and self-management programs. Patients can use this platform individually or together with their healthcare professional [19]. The aim was to interview fifteen to twenty patients until data saturation was achieved. Patients were eligible if they had a self-reported diagnosis of post-COVID according to the WHO-

For peer review only

For peer review only - http://bmjopen.bmj.com/site/about/guidelines.xhtml

137 definition, completed the ABCC-tool within the past three months to minimize recall bias, and
138 were proficient in the Dutch language. Before the start of the interviews, patients were
139 specifically asked whether they could remember completing the questionnaire and the balloon
140 diagram, and whether they had post-COVID. Patients were excluded if they could not
141 remember using the tool or seeing the balloon diagram. Patients were invited in small batches
142 to account for potential non-responses and scheduling conflicts.

143 ***Data collection***

144 Data were collected through single individual semi-structured online interviews in Dutch,
145 conducted between May and August 2024 by one female researcher (VHJD), who was trained
146 in qualitative research. All other researchers involved had experience in performing qualitive
147 research. Relevant topics for the interview were outlined in a topic guide developed by the
148 research team, which was pilot tested during the first interview. This topic guide was iteratively
149 adjusted between interviews to address emerging themes to enhance data richness, such as
150 the order of the questions. The final topic list is provided in Appendix 2. The two main topics
151 included the content validity of the ABCC-tool and its usability. Prior to the interviews, patients
152 received a printed copy of the tool's questionnaire and the balloon diagram by mail to recall
153 the tool and facilitate discussion. All interviews were audio-recorded with patients' consent. No
154 field notes were made during the interviews. The interviewer had no relationship with any of

For peer review only - http://bmjopen.bmj.com/site/about/guidelines.xhtml

the patients. Patients only were familiar with the aim of the research. The interviewer was introduced to the participants as a health scientist and researcher at Maastricht University.

Data analysis

Audio-recordings were subsequently transcribed using a custom implementation of WhisperX, which provides audio transcription based on Whisper (large-v2 model) and speaker diarization. This transcription process was performed locally on Maastricht University infrastructure/hardware [20]. Transcripts were 100% checked for correctness. Data analysis was conducted using a thematic approach with Atlas.ti version 23, software for qualitative data analysis. To analyze the transcripts, we used inductive – the process to identify new themes from textual data – and deductive – the process of applying pre-established codes to the transcripts – coding [21]. New themes were systematically monitored in between interviews to observe for data saturation and information power. Related codes were grouped into broader categories, and overarching themes were synthesized to gain a complete understanding of the patients' perspectives.

This iterative thematic analysis was reviewed and discussed with all members of the research team until consensus was reached. Initially, two researchers (VHJD and LHLP) independently coded 100% and 50% of all transcripts, respectively, to identify initial codes. The coding was

For peer review only - http://bmjopen.bmj.com/site/about/guidelines.xhtml

compared, and discrepancies were resolved through discussion, enhancing inter-rater reliability.

Patient and public involvement

No patients or public representatives were involved in any part of this study.

Results

In total, 5615 patients were invited for an interview, of which 27 interviews were started and 19 were completed and included in the analysis. Figure 2 presents the process of selecting and including patients. Ten men and nine women participated with a mean age of 56. Furthermore, approximately half of the patients had comorbidities. Eleven patients had a high educational level. No patients with a low educational level were included. In Table 1 the details per patient are presented. The length of the interviews ranged from 20 to 56 minutes. Data saturation was achieved for all topics, except comprehensiveness, after sixteen interviews. In no interview, additional people were present. One interview (interview 11) was cut short early due to concentration issues.

[INSERT FIGURE 2 HERE]

Figure 2: flowchart of the inclusion of patients for an interview

For peer review only - http://bmjopen.bmj.com/site/about/guidelines.xhtml

Table 1: Characteristics of the patients

Patient nr.	Age *	Gender	Other chronic conditions	Educational level**
1	Mid 50s	Female	Apnea<br>Lymphedema<br>Thyroid disorder	Medium
2	Late 50s	Female	-	High
3	Early 60s	Female	Asthma<br>Eczema	Medium
4	Early 50s	Female	Asthma	High
5	Early 60s	Male	Musculoskeletal disorder<br>Thyroid disorder	High
6	Mid 50s	Female	-	Medium
7	Late 50s	Female	-	High
8	Late 70s	Male	Apnea<br>Cardiovascular disease<br>Rheumatic disease	High
9	Early 50s	Male	Asthma	Medium
10	Early 50s	Male		Medium
11	Early 40s	Male	-	Medium
12	Mid 60s	Male	-	Medium
13	Late 50s	Female	-	High
14	Mid 50s	Male	COPD***	Medium
15	Late 40s	Male	-	High
16	Early 40s	Female	-	High
17	Early 50s	Male	-	High
18	Late 50s	Male	Rheumatic disease	High
19	Mid 60s	Female	-	High

*Age categories: early: age ending on 0-1-2-3, middle: 4-5-6, late: 7-8-9

**Dutch educational levels: *Low* = primary school and preparatory vocational secondary education (VMBO); *Medium* = senior general secondary education (HAVO), university preparatory education (VWO) and secondary vocational education and training (MBO); *High* = higher professional education (HBO) and research-oriented education (WO)[22]

*** COPD = chronic obstructive pulmonary disease

For peer review only - http://bmjopen.bmj.com/site/about/guidelines.xhtml

General evaluation of the ABCC-tool

Patients appreciated the tool's questionnaire and balloon diagram for providing a clear overview of both their current and past health status. The diversity of questions was also highly valued, especially those topics that were not typically linked to post-COVID by their healthcare professionals.

Patient 12: "I think it's a good tool. I believe it provides a lot of people with valuable insights into certain complaints, which might be related after all, things you wouldn't always immediately link, but that could indeed be connected in some way."

Several patients found it challenging to complete a questionnaire about their health over the past two weeks. They noted having both good and bad days, which made it difficult to answer the questions consistently. Some patients also struggled to remember their health status over the previous two weeks or from their last use of the tool. To address these challenges, some patients suggested incorporating extra text boxes to allow for personal notes.

Patient 13: "And what might be helpful […] is if there were spaces to write something down about your situation at that moment. […] For example, if I

For peer review only

For peer review only - http://bmjopen.bmj.com/site/about/guidelines.xhtml

look at my balloons now, I think, yeah, I have no idea what I was doing then, where I was, or if I was doing different things."

Most patients had used the ABCC-tool multiple times or expressed a willingness to use it more frequently. Nonetheless, many reported forgetting about the tool and suggested receiving periodic reminders, such as monthly or quarterly emails. Others mentioned they would only consider using the tool again if their symptoms changed.

Patient 11: "I think if I received an email saying it's been three months since you filled in the ABCC-tool. Click here to fill it in again, I would be more inclined to do it."

Seven patients required a more detailed explanation of how the tool worked, or what to do with it.

Patient 3: "I see the balloons, but I'm not really sure what I'm supposed to do with them."

Usability of the ABCC-tool

Not all patients could recall exactly where they found the ABCC-tool, but most patients had come across the ABCC-tool during online searches aimed at determining whether their symptoms and disease burden aligned with post-COVID. Others were alerted through

For peer review only - http://bmjopen.bmj.com/site/about/guidelines.xhtml

223 Facebook support groups, an email from C-support (a foundation of the Dutch ministry of

224 Health, Welfare, and Sport that supports and advises people with post-COVID), or by chance

225 through a Google search for treatments.

226 *Patient 18: "C-support, I believe. I get a lot from them because once you're*

227 *connected with them and that sort of things, you receive all kinds of*

228 *information, so to speak."*

229 Nobody experienced any difficulties with the practical use of the ABCC-tool. The website and

230 login process did not present any problems either.

231 *Patient 6: "No, I found it [the website] quite easy."*

232 The aim of the ABCC-tool was not clear to everyone. Some patients thought it was part of a

233 larger study to find an effective treatment for post-COVID, and that their participation would

234 contribute to the development of new treatments or pharmaceutical interventions. However,

235 most patients understood the aim of the tool.

236 *Patient 13: "I thought it was also for a broader study. I didn't realize it was*

237 *just for myself and possibly a healthcare provider. I actually didn't know*

238 *that."*

For peer review only - http://bmjopen.bmj.com/site/about/guidelines.xhtml

Most patients expressed a willingness to recommend the tool to other post-COVID patients, although some indicated they did not know any other post-COVID patients. One patient mentioned they would only recommend the tool if additional medical support would follow afterwards. Furthermore, two patients expressed a preference for more medical support after completing the ABCC-tool.

Patient 17: "It needs to be monitored. But if someone is consistently red month after month, then something is really wrong. And at that point, someone should be guided, like, hey, I think you need to be doing something else than what you're doing now. […] Or maybe referred to someone else."

Almost all patients found the questionnaire manageable in terms of length, as it could be paused and resumed at any time. However, one patient mentioned a lack of concentration to complete it and found the questionnaire too long.

Patient 7: "Yes, it was a nice experience. And I definitely liked that it can be saved in between. So, you don't have to complete it all at once. I did it in two parts."

For peer review only

For peer review only - http://bmjopen.bmj.com/site/about/guidelines.xhtml

Content of the ABCC-tool

The questions in the questionnaire were clear to all interviewed patients.

> *Patient 2: "Otherwise, I find it very clear. The language is very clear, and also how you rate [the answer from 0-6] it, so to speak, or the choices you have. Yes, it's just very clear to me."*

Regarding the comprehensiveness of the tool's questionnaire, data saturation was not achieved during the interviews. However, patients agreed that the most burdensome and most frequent burdens are included into the questionnaire. Additionally, four patients suggested including open-text fields throughout the questionnaire, allowing respondents to add personal notes about their current health status. This would facilitate easier comparison of responses across assessments. Another patient proposed incorporating a multiple-choice question to document any changes in areas such as health status, work, family situation, or other relevant personal factors.

Additionally, patients recommended adding questions regarding the type of therapy undergone, general pain experiences, balance difficulties, weight fluctuations due to edema, limitations in daily routines, symptom duration, feelings of being understood by government agencies, and restrictions experienced after intense physical activity.

For peer review only

For peer review only - http://bmjopen.bmj.com/site/about/guidelines.xhtml

Several suggestions were made to split questions that contain multiple items into separate questions. For example, two patients recommended breaking down the question on low, moderate, and vigorous physical activities into smaller steps to enable the possibility of more green balloons.

All questions were deemed relevant, except for one regarding sexuality, which was considered inappropriate by some in case of sharing the balloon diagram with their employer. Another patient felt this question was irrelevant for older individuals.

Patient 18: "I can imagine that the topic of sexuality in such a chart... it's not very convenient if you want to show it to your boss. That's not something they should be involved in."

The answer options were well-received, with most patients finding both the wording and number of response choices suitable.

Patient 11: "I think 0 to 6 is good. Actually, very good."

Visual representation

Most patients found the colored balloons easy to understand and fun to use. Furthermore, the balloon diagram made it easier to provide the patient with helpful insights into their health status. However, some patients were confused about whether the height of the balloons (e.g.

For peer review only

For peer review only - http://bmjopen.bmj.com/site/about/guidelines.xhtml

low balloons indicating low burden) or the colors (e.g. green balloon indicating low burden) indicated the burden of disease or were uncertain about the grey balloons and required further clarification. Those who had difficulty understanding the balloons suggested adding a legend or marking 'high' and 'low' burdens of disease on the vertical axis. Another patient expressed interest in seeing more long-term data, e.g. results from the past six or seven assessments.

Patient 12: "At least putting something down like high-low or a lot or not. […] That would have been much clearer, yes."

All patients recognized their health status in the balloon representation. While some appreciated the simplicity of the balloons for providing a clear overview of their current situation, others found the abundance of red balloons confronting. Furthermore, patients varied in their level of acceptance regarding their situation, with some demonstrating greater acceptance than others.

Patient 2: "It was really confronting for me, but it was clear. You really see it there, that was very helpful for me, to really tell myself […] it's really real."

Some patients shared the balloon diagram with healthcare professionals to discuss their current and past health, although not all healthcare professionals expressed interest in the balloon diagram. Those who preferred to use the ABCC-tool and balloon diagram together with

For peer review only - http://bmjopen.bmj.com/site/about/guidelines.xhtml

a healthcare professional would like to use it during visits with GPs, physical therapists, occupational therapists, rehabilitation specialists, or company doctors. While not everyone considered discussing the diagram with a healthcare professional, some patients expressed an intention to do so in the future. Others shared the balloon diagram with family, friends, or colleagues to explain their experiences with post-COVID. However, some patients felt they did not need the diagram, as they were able to explain their condition effectively without it. One patient found the diagram too confronting to share with others.

Patient 1: "No, I never showed the balloons. But I do know where my stumbling blocks are and what I need to work on."

Patient 6: "Yes, with my family members, my children. […] I find it nice to explain to them why I can't do certain things at times. Why I'm occasionally a bit down. […] They also understand it better."

Discussion

This study aimed to assess patients' perspectives on using the ABCC-tool for post-COVID, and the content validity of the questionnaire within the tool. As patients are experiential experts in their disease, in-depth interviews with them provided meaningful insights, emphasizing

For peer review only - http://bmjopen.bmj.com/site/about/guidelines.xhtml

essential elements for the tool’s use. The ABCC-tool was regarded as user-friendly and provided a comprehensive overview of the post-COVID burden.

Findings from this study confirm that the tool includes the most commonly reported post-COVID burdens and offers patients the opportunity to document additional concerns through an open-text feature. Furthermore, patients appreciated the tool for multiple reasons, meaning that the tool appears useful. First, the diversity of topics included in the tool, particularly as many of these are not routinely addressed in their healthcare. Second, patients found the tool's language accessible, because of its short sentences and simple wording [23]. Third, the length of the questionnaire was acceptable. Fourth, the visualization gave an insightful overview of disease burden. Finally, the tool was easy to find and use online.

For a few patients, the aim of the tool, and this study, was unclear and thought that the tool was part of a larger study to find a therapy or cure for post-COVID. This highlights how easily PROMs can be misunderstood, with potential consequences for engagement and expectations. To prevent such misperceptions, it is important that future use of the ABCC-tool is accompanied by clear communication about its purpose as a self-management and shared decision-making instrument, rather than as a diagnostic or therapeutic tool.

Since the emergence of post-COVID, several PROMs have been developed and validated to assess the disease burden, on functional status, symptom burden, QoL, and post-COVID

For peer review only

For peer review only - http://bmjopen.bmj.com/site/about/guidelines.xhtml

related stigma [24]. Examples of tools are the Symptom Burden Questionnaire for Long COVID (SBQ-LB), the Post COVID-19 Condition Stigma Questionnaire (PCCSQ), and the Long COVID Symptoms and Severity Score (LC-SSS scale) [25-28]. When comparing these tools to the ABCC-tool, we concluded that the ABCC-tool (44 items) is similar in length compared with the LC-SSS (44 items) and PCCSQ tool (40 items), with all three being scored on a 4 to 7 point Likert scale [25-28]. However, the ABCC-tool stands out, as none of these other tools specifically focus on the core dimensions of disease burden as defined by the WHO (i.e., physical, emotional, and social burden) and incorporate a visual representation of the results. It is important, however, to note that while these tools differ in scope and structure, no formal comparison was conducted regarding user experience or completion time. Therefore, although patients rated the ABCC-tool as user-friendly in this study, no conclusions can yet be drawn about its relative usability or efficiency compared to other available post-COVID PROMs. Future studies could explore these aspects in more depth through head-to-head comparisons.

In the current study, patients specifically preferred this visual representation. Although the balloon diagram was not immediately clear to everyone, a brief explanation made it fully understandable. Therefore, the inclusion of a legend for the balloon diagram was considered essential. This is congruent with recommendations that have been made in studies with the

For peer review only

ABC-, ABCC-, and Assessment of Burden of ColoRectal Cancer (ABCRC-) tool, to facilitate better understanding of the diagram [12,29,30]. The balloons gave patients understandable insights into their health status, facilitating structured discussions with healthcare professionals. However, some patients had numerous red balloons which felt confronting and negatively impacted on their motivation, for example, to adopt a healthier lifestyle. It is important to note that many of these patients had comorbidities, making it difficult to determine whether their reported burdens were due to post-COVID or these pre-existing conditions. Therefore, incorporating post-COVID into the ABCC-tool is more beneficial than developing a stand-alone tool, as the ABCC-tool includes multiple chronic conditions in a single PROM by integrating the relevant modules for each condition [9-11].

Additionally, other adjustments were recommended to make the tool more suitable for use, these include, amongst others, more open-text fields to accommodate personal notes. Given that memory loss and cognitive impairment are well-documented burdens of post-COVID (2), patients often struggle to recall their experiences from previous weeks. This leads to difficulties in distinguishing day-to-day changes. Therefore, patients preferred the option to make notes to ensure more accurate responses. Furthermore, both patients in this study and those utilizing the ABC-, ABCC-, and ABCRC-tool have suggested the implementation of periodic email reminders to encourage more frequent completion of the questionnaire [12,29,30]. Research

For peer review only

For peer review only - http://bmjopen.bmj.com/site/about/guidelines.xhtml

indicates that sending email reminders can significantly improve the completion rate of PROMs (22). Patients indicated that the tool would be more relevant if they had more frequent follow-up results to better monitor their progress.

Only limited evidence is available on optimal treatment strategies for post-COVID, as it is a relatively new disease. Currently, most treatment programs involve wait-and-see approaches, physical exercise, and lifestyle recommendations [31,32]. Ideally, post-COVID care should move towards a more personalized and holistic approach. However, this is challenging due to the lack of robust evidence on what interventions are effective per symptom, the high number of reported symptoms, and the substantial variability in burden among individuals [2,5,6]. Given these uncertainties, the ABCC-tool could play a role in addressing this gap.

Strengths and limitations

This study has several strengths and limitations. The qualitative study design provided in depth results in the content validation and the perspective of patient of the ABCC-tool. The use of both inductive and deductive approaches strengthened the study's credibility. The inductive coding process minimized confirmation bias by allowing themes and patterns to naturally emerge from the data, rather than being predefined. Meanwhile, deductive analysis reduced researcher bias by cross-verifying themes with existing frameworks [33-36].

For peer review only

For peer review only - http://bmjopen.bmj.com/site/about/guidelines.xhtml

Data saturation was not fully achieved on comprehensiveness, however, we systematically monitored whether new symptoms or themes emerged during the interviews. While some additional complaints were mentioned, these largely overlapped with issues already identified, or were not applicable for the ABCC-tool. We therefore argue that information power was sufficient, as the sample provided rich and relevant data on the clarity and relevance of the questionnaire items. Factors such as the study's aim and the quality of the collected data play an important role in determining whether the sample holds sufficient information for meaningful analysis [37]. Due to the complexity of post-COVID and the aforementioned over 200 symptoms that are associated with this condition [2], it is not feasible to include all symptoms in the questionnaire. Furthermore, the included symptoms reflect the most prevalent burdens described in the literature on post-COVID, such as fatigue and cognitive dysfunction, which were consistently confirmed by our participants [1,2,4]. We therefore decided that this issue can only be solved by incorporating an open question asking complaints other than those already mentioned. This way, the most prevalent burdens are included into the tool, with space for additional burdens to report.

In total, only 143 of the 5615 invited patients were eligible and interested in participating. We suspect that concentration problems commonly associated with post-COVID may have discouraged many patients from completing the interviews, as these were perceived as too

For peer review only

For peer review only - http://bmjopen.bmj.com/site/about/guidelines.xhtml

411 demanding, amongst others. This may have led to selection bias: those who volunteer to
412 participate in scientific research are mostly woman, higher educated, and score higher on
413 cognitive tests [38]. The underrepresentation of patients with a low educational level could
414 potentially limit the external validity of the findings.

415 We interviewed a roughly equal number of men (n=10) and women (n=9), including patients
416 with and without comorbidities. However, we were not able to take into account educational
417 level when including patients, as this information was unavailable beforehand. Despite the
418 random selection no participants with lower educational levels were included. Regardless, we
419 expect the tool's questions to be accessible and readable for individuals with lower education
420 due to their B1 language level. Furthermore, previous studies on the ABCC tool's content
421 validity and patient experiences included participants with both higher and lower educational
422 levels [10,12]. None of the patients reported difficulties with using the tool or understanding its
423 language. Although the current study focused on a different patient population, the tool's
424 purpose, functioning, and linguistic level remain unchanged. For future research, we
425 recommend collecting basic patient information to enable more targeted recruitment and
426 selection.

427 Our inclusion criteria were not objectively verifiable. First, we could not confirm whether the
428 patient had post-COVID or not, as there is no standardized or validated diagnostic method for

For peer review only - http://bmjopen.bmj.com/site/about/guidelines.xhtml

429 this condition available. Currently, diagnoses are typically made by healthcare professionals or self-reported by patients, based on a set of symptoms. Second, it was impossible to verify the timing of patients' last use of the tool online, as these data were not accessible by the research team. To address these limitations, patients were asked at the start of the interview to specify who provided their post-COVID diagnosis and elaborate when this was self-diagnosed. They were also asked to indicate when they last used the ABCC-tool and whether they could remember this to minimize recall bias. If either of these two criteria was not met, the interview was terminated. For some patients, lack of concentration was a significant burden of post-COVID, which impacted their ability to participate in lengthy interviews. One interview had to be cut short on the demand of the patient. Additionally, some patients mentioned at the beginning of the interview not to be able to talk longer than a specific time. In that case, the interviewer started with the most important topics of the study (i.e. usability of the tool and content validation of the questionnaire). For the same reason, transcripts were not returned to the patients because this would be too burdensome. However, we acknowledge that offering participants the option to review their transcripts could have provided an additional opportunity for validation and feedback, and we will consider this approach in future studies.

For peer review only - http://bmjopen.bmj.com/site/about/guidelines.xhtml

Implications for future research and practice

A few adjustments (open text fields, periodic reminders, including a figure legend, and clarifying the purpose of the tool) need to be made to the tool, based on the findings in this research. Furthermore, although the questions of the tool have now been validated, a thorough evaluation of the other aspects of the tool is in place. For example, the recommended score calculation and lifestyle advice regarding physical activity might not be appropriate in the case of post-exertional malaise (PEM). This needs further investigation.

After these adjustments, and further evaluation of the scores, a pilot study should be conducted to evaluate the usability and feasibility of the tool in healthcare settings. This study should include interviews with patients and healthcare professionals to evaluate the barriers and facilitators for using the tool, and study the usability from different perspectives. Additionally, evidence-based treatment recommendations for each domain must be developed and integrated into the tool before clinical implementation. Technical aspects, such as seamless integration into electronic health records, should also be addressed to ensure its practical use in healthcare systems. These steps would enable the ABCC-tool to enhance care for post-COVID patients.

Given the positive results of the ABCC-tool in assessing disease burden in patients with post-COVID, further research is needed to explore its implementation in clinical practice. As post-

For peer review only - http://bmjopen.bmj.com/site/about/guidelines.xhtml

464 COVID is a relatively new condition, the first step is to establish guidelines supporting person-
465 centered care and shared decision making. The ABCC-tool could play a key role in supporting
466 this by providing a structured and efficient holistic assessment of patients' burden and needs.

467 Although the ABCC-tool was originally developed in the Netherlands, future research could
468 explore its implementation in other countries. This would require careful translation and cultural
469 validation to ensure that the tool is both linguistically accurate and contextually appropriate for
470 use in different healthcare settings. The first interest in the ABCC-tool of other countries has
471 been reported already. Furthermore, it may be valuable to explore the potential application of
472 the ABCC tool for post-COVID in patients with myalgic encephalomyelitis/chronic fatigue
473 syndrome (ME/CFS), as the burden associated with ME/CFS closely resembles that of post-
474 COVID. Both conditions are characterized by substantial health, economic, and social impacts,
475 which are often underestimated due to limited awareness and understanding of these diseases
476 [39].

477

478 *Conclusion*

479 The ABCC-tool is seen as a useful tool to assess experienced burden of disease in patients
480 with post-COVID and to monitor the course of symptoms in time. The most appreciated aspects
481 of the tool are the balloon diagram, which presents the current and past health situation of the

For peer review only - http://bmjopen.bmj.com/site/about/guidelines.xhtml

patient, and the diverse range of questions in the questionnaire. Furthermore, the content of the questionnaire is praised for being comprehensive, comprehensible, and relevant for post-COVID. Based on the current and previous studies on the ABCC-tool, the tool for post-COVID holds significant potential to enhance person-centered care for post-COVID patients and is suitable for integration into clinical practice.

Declarations

Funding and competing interests

This study was funded by ZonMw (10430302130003) and Pfizer (75242181). The ZonMw grant was received by Jean Muris, with JSB as project leader. The grant of Pfizer was received by AHMGS. More information is available on https://www.pfizer.nl/. The funders had no role in study design, data collection and analysis, decision to publish, or preparation of the manuscript. All other authors have no completing interest to declare.

Authors' contributions

VHJD, RPGO, and AHMGS were involved in the study design. VHJD conducted the interviews. VHJD and LHLP performed the coding. All authors reviewed the thematic analysis and reached a consensus. Furthermore, all authors contributed to the writing process, provided feedback, and approved the final version of the manuscript.

For peer review only

Ethics approval and consent to participate

Ethical approval was provided by the Medical Ethics Committee of the Maastricht University Medical Centre (METC 2022-3439). All interviewed patients provided written informed consent before the interview.

Availability of data and material

Data will be stored in a repository called DataHub. The DOI will become available shortly.

Acknowledgement

We would like to thank all the patients who showed interest in participating in this study. Furthermore, we would like to thank CuraVista for their contribution in the development and implementation of the ABCC-tool. In addition, this research was made possible, in part, using the Data Science Research Infrastructure (DSRI) hosted at Maastricht University.

For peer review only

References

1. Cha C, Baek G. Symptoms and management of long COVID: A scoping review. Journal of Clinical Nursing. 2024;33(1):11-28.
2. World Health Organization. Post COVID-19 codition (Long COVID) 2022. Available from: https://www.who.int/europe/news-room/fact-sheets/item/post-covid-19-condition
3. World Health Organization. Post COVID-19 condition (long COVID) 2025.
4. Frallonardo L, Ritacco AI, Amendolara A, et al. Long-Term Impairment of Working Ability in Subjects under 60 Years of Age Hospitalised for COVID-19 at 2 Years of Follow-Up: A Cross-Sectional Study. Viruses. 2024;16(5):688.
5. Bonilla H, Peluso MJ, Rodgers K, et al. Therapeutic trials for long COVID-19: A call to action from the interventions taskforce of the RECOVER initiative. Frontiers in immunology. 2023;14:1129459.
6. Greenhalgh T, Sivan M, Delaney B, et al. Long covid—an update for primary care. bmj. 2022;378.
7. Platteel TN, Koelmans JC, Cianci D, et al. Long-term prognosis of adults with moderately severe SARS-CoV-2 lower respiratory tract infection managed in primary care: Prospective cohort study. Eur J Gen Pract. 2025 Dec;31(1):2501306.
8. van Noort EMJ, Claessens D, Moor CC, et al. Online Tool for the Assessment of the Burden of COVID-19 in Patients: Development Study. JMIR Form Res. 2021 Mar 31;5(3):e22603.
9. Boudewijns EA, Claessens D, van Schayck OC, et al. ABC-tool reinvented: development of a disease-specific 'Assessment of Burden of Chronic Conditions (ABCC)-tool'for multiple chronic conditions. BMC family practice. 2020;21(1):1-7.
10. Debie VHJ, Boymans TAEJ, Ottenheijm RPG, et al. Expanding the ABCC-tool for Osteoarthritis: Development and Content Validation. Osteoarthritis and Cartilage Open. 2024;6(3):100488.
11. Slok AHM, Chavannes NH, van der Molen T, et al. Development of the Assessment of Burden of COPD tool: an integrated tool to measure the burden of COPD. NPJ Primary Care Respiratory Medicine. 2014;24(1):1-4.
12. Slok AH, Twellaar M, Jutbo L, et al. 'To use or not to use': a qualitative study to evaluate experiences of healthcare providers and patients with the assessment of burden of COPD (ABC) tool. npj Primary Care Respiratory Medicine. 2016;26(1):1-8.

For peer review only - http://bmjopen.bmj.com/site/about/guidelines.xhtml

13. Europass. Het gemeenschappelijk Europees referentiekader voor talen n.d. Available from: https://europass.europa.eu/nl/common-european-framework-reference-language-skills

14. Boudewijns EA, Claessens D, van Schayck OC, et al. Effectiveness of the Assessment of Burden of Chronic Conditions (ABCC)-tool in patients with asthma, COPD, type 2 diabetes mellitus, and heart failure: A pragmatic clustered quasi-experimental study in the Netherlands. European Journal of General Practice. 2024;30(1):2343364.

15. Tindall L. J.A. Smith, P. Flower and M. Larkin (2009), Interpretative Phenomenological Analysis: Theory, Method and Research. Qualitative Research in Psychology. 2009 2009/11/25;6(4):346-347.

16. Pietkiewicz I, Smith JA. A practical guide to using interpretative phenomenological analysis in qualitative research psychology. Psychological journal. 2014;20(1):7-14.

17. Terwee CB, Prinsen C, Chiarotto A, et al. COSMIN methodology for assessing the content validity of PROMs–user manual. Amsterdam: VU University Medical Center. 2018.

18. Tong A, Sainsbury P, Craig J. Consolidated criteria for reporting qualitative research (COREQ): a 32-item checklist for interviews and focus groups. Int J Qual Health Care. 2007 Dec;19(6):349-57.

19. van Zelst CM, Kasteleyn MJ, van Noort EMJ, et al. The impact of the involvement of a healthcare professional on the usage of an eHealth platform: a retrospective observational COPD study. Respir Res. 2021 Mar 21;22(1):88.

20. Radford A, Kim JW, Xu T, et al., editors. Robust speech recognition via large-scale weak supervision. International conference on machine learning; 2023: PMLR.

21. Chandra Y, Shang L. Inductive Coding. In: Chandra Y, Shang L, editors. Qualitative Research Using R: A Systematic Approach. Singapore: Springer Nature Singapore; 2019. p. 91-106.

22. CBS. Opleidingsniveau. Available from: https://www.cbs.nl/nl-nl/nieuws/2019/33/verschil-levensverwachting-hoog-en-laagopgeleid-groeit/opleidingsniveau

23. Ministerie van Algemene Zaken. Taalniveau B1. Available from: https://www.communicatierijk.nl/vakkennis/rijkswebsites/aanbevolen-richtlijnen/taalniveau-b1

24. Baalmann A-K, Blome C, Stoletzki N, et al. Patient-reported outcome measures for post-COVID-19 condition: a systematic review of instruments and measurement properties. BMJ Open. 2024;14(12):e084202.

For peer review only

For peer review only - http://bmjopen.bmj.com/site/about/guidelines.xhtml

25. Hughes SE, Haroon S, Subramanian A, et al. Development and validation of the symptom burden questionnaire for long covid (SBQ-LC): Rasch analysis. Bmj. 2022 Apr 27;377:e070230.
26. Damant RW, Rourke L, Cui Y, et al. Reliability and validity of the post COVID-19 condition stigma questionnaire: A prospective cohort study. EClinicalMedicine. 2023 Jan;55:101755.
27. Ye G, Zhu Y, Bao W, et al. The Long COVID Symptoms and Severity Score: Development, Validation, and Application. Value Health. 2024 Aug;27(8):1085-1091.
28. Tran VT, Riveros C, Clepier B, et al. Development and Validation of the Long Coronavirus Disease (COVID) Symptom and Impact Tools: A Set of Patient-Reported Instruments Constructed From Patients' Lived Experience. Clin Infect Dis. 2022 Jan 29;74(2):278-287.
29. Thomassen BJ, Gielen AH, Baak J, et al. Supporting follow-up care in colorectal cancer patients: first experiences with the Assessment of Burden of ColoRectal Cancer (ABCRC)-tool. Journal of Cancer Survivorship. 2024:1-11.
30. Peters LHL, Keijsers LCEM, van Schayck OCP, et al. Patient Experiences with the Assessment of Burden of Chronic Conditions (ABCC) Tool in Primary Care: A Qualitative Study. Submitted.
31. Bachmeier BE, Hölzle S, Gasser M, et al. How do German general practitioners manage long-/post-COVID? A qualitative study in primary care. Viruses. 2023;15(4):1016.
32. Kogel A, Machatschek M, Scharschmidt R, et al. Physical exercise as a treatment for persisting symptoms post-COVID infection: Review of ongoing studies and prospective randomized controlled training study. Clinical Research in Cardiology. 2023;112(11):1699-1709.
33. Fereday J, Muir-Cochrane E. Demonstrating rigor using thematic analysis: A hybrid approach of inductive and deductive coding and theme development. International journal of qualitative methods. 2006;5(1):80-92.
34. Hsieh H-F, Shannon SE. Three approaches to qualitative content analysis. Qualitative health research. 2005;15(9):1277-1288.
35. Nickerson RS. Confirmation bias: A ubiquitous phenomenon in many guises. Review of general psychology. 1998;2(2):175-220.
36. Nowell LS, Norris JM, White DE, et al. Thematic analysis: Striving to meet the trustworthiness criteria. International journal of qualitative methods. 2017;16(1):1609406917733847.

For peer review only

For peer review only - http://bmjopen.bmj.com/site/about/guidelines.xhtml

37. Malterud K, Siersma VD, Guassora AD. Sample size in qualitative interview studies: guided by information power. Qualitative health research. 2016;26(13):1753-1760.

38. Tripepi G, Jager KJ, Dekker FW, et al. Selection bias and information bias in clinical research. Nephron Clinical Practice. 2010;115(2):c94-c99.

39. Vester P, Boudouroglou-Walter S, Schreyögg J, et al. Burden of Disease in Myalgic Encephalomyelitis/Chronic Fatigue Syndrome (ME/CFS): A Scoping Review. Appl Health Econ Health Policy. 2025 Sep 23.

For peer review only

For peer review only - http://bmjopen.bmj.com/site/about/guidelines.xhtml

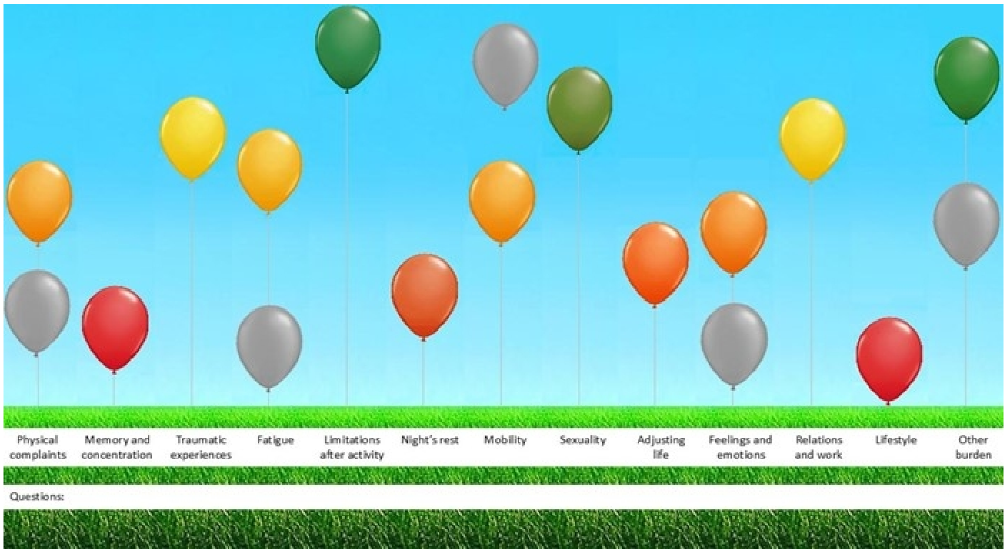

Figure 1: The balloon diagram of the ABCC-tool for post-COVID. This diagram presents the results of the questionnaire from the ABCC-tool. Red balloons indicate a high burden of disease, while green balloons represent a low burden. Yellow and orange balloons depict intermediate scores, and grey balloons show the scores from the previous completion of the ABCC-tool.

185x101mm (96 x 96 DPI)

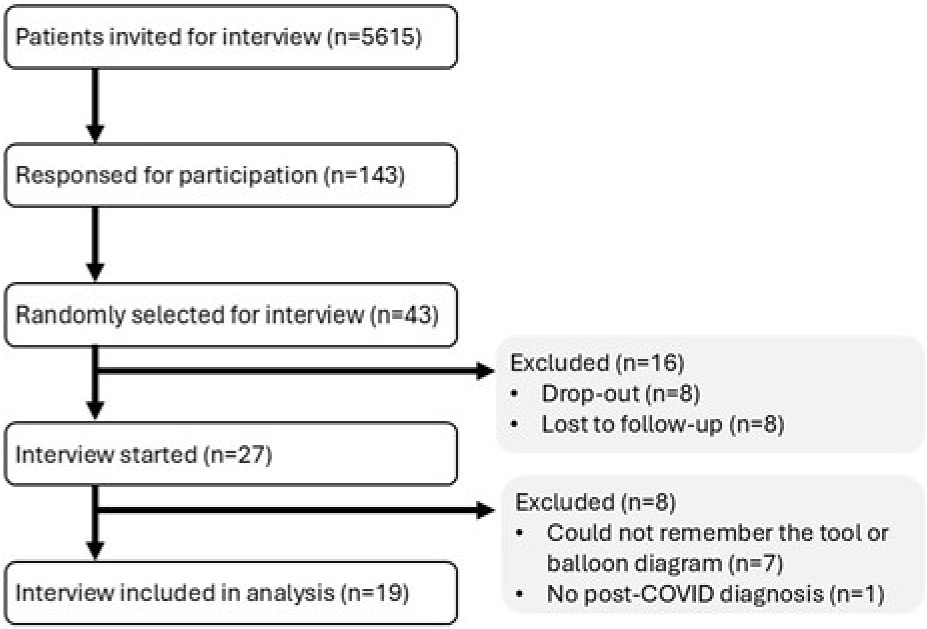

Figure 2: flowchart of the inclusion of patients for an interview

127x85mm (96 x 96 DPI)

APPENDIX 1: QUESTIONNAIRE IN THE ABCC-TOOL

ENGLISH VERSION

(Note: this is an unvalidated translation from Dutch to English specifically for this paper)

Dear Sir/Madam,
With this questionnaire, we would like to get an impression of how you are doing. During consultation with your healthcare provider, you can talk about the topics that are important to you.
These questions are related to the chronic condition(s) for which you are visiting the healthcare provider.

What were you suffering from?

	In the past __week__, how often ….	0 Never	1 Hardly ever	2 A few times	3 Several times	4 Many times	5 A great many times	6 Almost all the time
1	did you suffer from **fatigue**?	☐	☐	☐	☐	☐	☐	☐
2	did you have a poor **night's rest**?	☐	☐	☐	☐	☐	☐	☐
3	did you suffer from sadness, fear, frustration, shame or other **unpleasant feelings**?	☐	☐	☐	☐	☐	☐	☐
4	did you suffer **adjusting your life** (e.g. planning of activities, get enough exercise, eating healthy)?	☐	☐	☐	☐	☐	☐	☐
	In the past 2 __weeks__, to what extend …	0 Not at all	1 Very slightly	2 Slightly	3 Moderate	4 Very	5 Extremely	6 Totally
5	did you suffer from a **decreased condition**?	☐	☐	☐	☐	☐	☐	☐
6	did you suffer from **pain/ difficulties with breathing**?	☐	☐	☐	☐	☐	☐	☐
7	did you suffer from **coughing**?	☐	☐	☐	☐	☐	☐	☐
8	did you suffer from **pain on the chest** or **a feeling of pressure on the chest**?	☐	☐	☐	☐	☐	☐	☐
9	did you suffer from **palpitations**?	☐	☐	☐	☐	☐	☐	☐
10	did you suffer from a **strange taste or smell, or tasted/smelled nothing**?	☐	☐	☐	☐	☐	☐	☐
11	did you suffer from **earache** or **tinnitus**?	☐	☐	☐	☐	☐	☐	☐
12	did you suffer from a **lump in your throat** or a **sore throat**?	☐	☐	☐	☐	☐	☐	☐
13	did you suffer from problems with **chewing and/or swallowing**?	☐	☐	☐	☐	☐	☐	☐
14	did you suffer from **heavy arms or legs**?	☐	☐	☐	☐	☐	☐	☐
15	did you suffer from **joint and/or muscle pain**?	☐	☐	☐	☐	☐	☐	☐
16	did you suffer from **tingling in your hands, feet, arms or legs**?	☐	☐	☐	☐	☐	☐	☐
17	did you suffer from **a headache**?	☐	☐	☐	☐	☐	☐	☐
18	did you suffer from **dizziness**?	☐	☐	☐	☐	☐	☐	☐
19	did you suffer from problems with **concentration and/or memory**?	☐	☐	☐	☐	☐	☐	☐

For peer review only - http://bmjopen.bmj.com/site/about/guidelines.xhtml

In the past 2 weeks, to what extend …		0<br>Not at all	1<br>Very slightly	2<br>Slightly	3<br>Moderate	4<br>Very	5<br>Extremely	6<br>Totally
20	did you feel **overstimulated**?	□	□	□	□	□	□	□
21	did you suffer from **bad memories, flashbacks or nightmares**?	□	□	□	□	□	□	□
22	did you suffer from **blurred and/or double vision, dry or painful eyes**?	□	□	□	□	□	□	□
23	did you suffer from **abdominal complaints**?	□	□	□	□	□	□	□
24	did you suffer from **feeling hot and cold alternately** or did you have a **fever**?	□	□	□	□	□	□	□
25	did you suffer from **dry or sore skin**?	□	□	□	□	□	□	□
26	did you suffer from **hair loss**?	□	□	□	□	□	□	□
In the past 2 weeks, …		Not	2-3 hours	4-10 hours	11-13 hours	14-24 hours	24-48 hours	Longer than 48 hours
27	if you **felt worse after exercising**, how long did this last?	□	□	□	□	□	□	□
28	did you suffer from **any complaints other than those mentioned above**?	I suffered from:						

In what did you feel limited?

In the past week, to what extend …		0<br>Not at all	1<br>Very slightly	2<br>Slightly	3<br>Moderate	4<br>Very	5<br>Extremely	6<br>Totally
29	were you limited in **strenuous physical** activities (such as climbing stairs, hurrying, doing sports)?	□	□	□	□	□	□	□
30	were you limited in **moderate physical** activities (such as walking, housework, carrying things)?	□	□	□	□	□	□	□
31	were you limited in **daily activities** at home (such as dressing, washing yourself)	□	□	□	□	□	□	□
32	were you limited in your **work** (paid word, unpaid work, volunteer work)?	□	□	□	□	□	□	□
33	were you limited in **moving around outside your home**?	□	□	□	□	□	□	□
34	Were you limited in your **social activities** (visiting friends and family, daytrips)?	□	□	□	□	□	□	□
35	had your condition a negative impact on your **relations with others**?	□	□	□	□	□	□	□
36	did you have any difficulty with **intimacy or sexuality**?	□	□	□	□	□	□	□
37	did you **worry about your future**?	□	□	□	□	□	□	□

For peer review only

How was your lifestyle?

38	In the past week, how many days have you had moderately intense **physical exercise** for 30 minutes or more?<br>E.g. walking or cycling in a fast pace<br>It may also be a minimum of 3x10 minutes	□ 0 days<br>□ 1-2 days<br>□ 3-4 days<br>□ 5 days or more
39	How many glasses of **alcohol** did you drink in the past week?	.... glasses per week
40	Do you **smoke** or have you smoked?	□ Yes. In the last week, how many (shag) cigarettes have you smoked on average per day? ____________<br>□ Previously. Stopped smoking since: ____ (month) / ____ (year)<br>□ Never
41	What is your **weight**?	.... kg
42	What is your **height**?	.... cm
43	In the past week, how many days did you **eat healthy**?	□ 0 days<br>□ 1-2 days<br>□ 3-4 days<br>□ 5 days or more
44	Is there anything else you would like to **discuss** or would like to receive more **information** about?<br>____<br>____	

For peer review only

DUTCH VERSION

Beste meneer/mevrouw,
Met deze vragenlijst willen we samen met u in kaart brengen hoe het met u gaat.
Deze vragen hebben te maken met de chronische aandoening(en) waarvoor u bij de zorgverlener komt.

Waar had u last van?

	In de afgelopen week, hoe vaak …	0 Nooit	1 Zelden	2 Af en toe	3 Regel-matig	4 Heel vaak	5 Meestal	6 Altijd
1	had u last van **vermoeidheid**?	☐	☐	☐	☐	☐	☐	☐
2	had u een slechte **nachtrust**?	☐	☐	☐	☐	☐	☐	☐
3	had u last van somberheid, angst, frustratie, schaamte of andere **vervelende gevoelens**?	☐	☐	☐	☐	☐	☐	☐
4	had u **moeite om uw leven aan te passen** (bijv. plannen van activiteiten, voldoende beweging, gezond eten)?	☐	☐	☐	☐	☐	☐	☐

	In de afgelopen 2 weken, in welke mate …	0 Hele-maal niet	1 Heel weinig	2 Een beetje	3 Tamelijk	4 Erg	5 Heel erg	6 Volledig
5	had u last van een **verminderde conditie**?	☐	☐	☐	☐	☐	☐	☐
6	had u last van **pijn of moeite met ademhalen**?	☐	☐	☐	☐	☐	☐	☐
7	had u last van **hoesten**?	☐	☐	☐	☐	☐	☐	☐
8	had u last van **pijn op de borst of een drukkend gevoel op de borst**?	☐	☐	☐	☐	☐	☐	☐
9	had u last van **hartkloppingen**?	☐	☐	☐	☐	☐	☐	☐
10	had u last van een **vreemde smaak of geur, of proefde/rook u niets**?	☐	☐	☐	☐	☐	☐	☐
11	had u last van **oorpijn of oorsuizen**?	☐	☐	☐	☐	☐	☐	☐
12	had u last van een **brok in uw keel of keelpijn**?	☐	☐	☐	☐	☐	☐	☐
13	had u last van **kauw- en/of slikproblemen**?	☐	☐	☐	☐	☐	☐	☐
14	had u last van **zware armen of benen**?	☐	☐	☐	☐	☐	☐	☐
15	had u last van **gewrichts- en/of spierpijn**?	☐	☐	☐	☐	☐	☐	☐
16	had u last van **tintelingen in uw handen, voeten, armen of benen**?	☐	☐	☐	☐	☐	☐	☐
17	had u last van **hoofdpijn**?	☐	☐	☐	☐	☐	☐	☐
18	had u last van **duizeligheid**?	☐	☐	☐	☐	☐	☐	☐
19	had u last van **concentratie- en/of geheugenproblemen**?	☐	☐	☐	☐	☐	☐	☐
20	had u het gevoel **overprikkeld** te zijn?	☐	☐	☐	☐	☐	☐	☐
21	had u last van **nare herinneringen, herbelevingen of nachtmerries**?	☐	☐	☐	☐	☐	☐	☐
22	had u last van **wazig en/of dubbelzien, droge of pijnlijke ogen**?	☐	☐	☐	☐	☐	☐	☐

For peer review only

	In de afgelopen 2 weken, in welke mate …	0<br>Helemaal niet	1<br>Heel weinig	2<br>Een beetje	3<br>Tamelijk	4<br>Erg	5<br>Heel erg	6<br>Volledig
23	had u last van **buikklachten**?	□	□	□	□	□	□	□
24	had u last van u zich **afwisselend warm en koud voelen of had u koorts**?	□	□	□	□	□	□	□
25	had u last van een **droge of pijnlijke huid**?	□	□	□	□	□	□	□
26	had u last van **haaruitval**?	□	□	□	□	□	□	□
	In de afgelopen 2 weken …	Niet	2-3 uur	4-10 uur	11-13 uur	14-24 uur	24-48 uur	langer dan 48 uur
27	als u zich **slechter voelde na het doen van een inspanning**, hoe lang hield dat dan aan?	□	□	□	□	□	□	□
28	had u last van **klachten anders dan die hierboven al genoemd zijn**?	Ik had last van:						

Waarin voelde u zich beperkt?

	In de afgelopen week, in welke mate …	0<br>Helemaal niet	1<br>Heel weinig	2<br>Een beetje	3<br>Tamelijk	4<br>Erg	5<br>Heel erg	6<br>Volledig
29	voelde u zich beperkt in **zware lichamelijke activiteiten** (trap lopen, haasten, sporten)?	□	□	□	□	□	□	□
30	voelde u zich beperkt in **matige lichamelijke activiteiten** (wandelen, huishoudelijk werk, boodschappen doen)?	□	□	□	□	□	□	□
31	voelde u zich beperkt in **dagelijkse activiteiten** (u zelf aankleden, wassen)?	□	□	□	□	□	□	□
32	voelde u zich beperkt in uw **werk** (betaald werk, onbetaald werk, vrijwilligerswerk)?	□	□	□	□	□	□	□
33	voelde u zich beperkt in het u **verplaatsen buitenshuis**?	□	□	□	□	□	□	□
34	voelde u zich beperkt in uw **sociale activiteiten** (uitjes, vrienden en familie bezoeken)?	□	□	□	□	□	□	□
35	had uw aandoening een negatieve invloed op uw **relatie met anderen**?	□	□	□	□	□	□	□
36	had u moeite met **intimiteit** of **seksualiteit**?	□	□	□	□	□	□	□
37	maakte u zich **zorgen over uw toekomst**?	□	□	□	□	□	□	□

For peer review only

Hoe ging het met uw leefstijl?

38	In de afgelopen week, hoeveel dagen heeft u 30 minuten of meer matig intensieve **lichaamsbeweging** gehad?<br>Bijv. stevig doorwandelen of harder fietsen.<br>Het mogen ook minimaal 3x10 minuten zijn.	□ 0 dagen<br>□ 1-2 dagen<br>□ 3-4 dagen<br>□ 5 dagen of meer
39	Hoeveel glazen **alcohol** dronk u in de afgelopen week?	… glazen per week
40	Rookt u of heeft u **gerookt**?	□ Ja. Hoeveel (shag)sigaretten heeft u in de afgelopen week gemiddeld per dag gerookt? ____________<br>□ Vroeger. Gestopt met roken sinds: ___(maand) / ___(jaar)<br>□ Nooit
41	Wat is uw **gewicht**?	………. kg
42	Wat is uw **lengte**?	………. cm
43	In de afgelopen week, hoeveel dagen heeft u **gezond gegeten**?	□ 0 dagen<br>□ 1-2 dagen<br>□ 3-4 dagen<br>□ 5 dagen of meer
44	Is er nog iets wat u wilt **bespreken** of waar u **meer informatie** over wilt krijgen?<br>____<br>____	

For peer review only

APPENDIX 2: TOPIC LIST (ORIGINALLY IN DUTCH)

Demographic questions

1. What is your age?
2. How is your health now?
    a. Do you have other chronic conditions?
3. How was your health before you had post-COVID?
4. What is your educational level?
5. What is your gender?

Acute COVID-19

6. When did you have acute COVID-19?
7. Were you admitted to the hospital?
    a. If yes: where you admitted to the intensive care?
8. How did you get the diagnosis?

Post-COVID

9. Since when do you have post-COVID symptoms?
10. By whom and how where you diagnosed with post-COVID

ABCC-tool

11. How did you discover the ABCC-tool?
12. When and how many times did you complete the ABCC-tool?
13. Would you like to use the ABCC-tool again in the future?
14. Why did you complete the ABCC-tool?
    a. What was your purpose with it?
    b. Did the ABCC-tool meet those purposes?

Content validation

15. Was the ABCC-tool complete (comprehensive)?
16. Was question ... relevant for you? (per item in the questionnaire)
17. Was question ... formulated clearly? (per item in the questionnaire)
18. What were your thoughts about the answer options?

Usability

19. How was the ABCC-tool online to use?
    a. Was it easy to log in on the app/website?
    b. Was the app/website clear?
20. How much time did it take?
21. What were your thoughts about the balloon diagram?
    a. Was it clear?
    b. Would you like to change anything about it?
22. Did you discuss the balloon diagram with somebody?
    a. If yes: with whom? And why?
23. In what way has the balloon diagram helped you explain how you feel?
    a. Did it give you more compassion from a healthcare professional?
    b. Did it give you more compassion from family or friends?
24. Would you recommend the ABCC-tool to others?
    a. If yes: to whom do you want to recommend the ABCC-tool?

For peer review only

BMJ Open

Patient perspectives on the usability and content validity of the Assessment of Burden of Chronic Conditions (ABCC-) tool for post-COVID in the Netherlands: a qualitative study

Journal:	*BMJ Open*
Manuscript ID	bmjopen-2025-109201.R2
Article Type:	Original research
Date Submitted by the Author:	18-Nov-2025
Complete List of Authors:	Debie, Valerie ; Maastricht University, Care and Public Health Research Institute (CAPHRI), Department of Family Medicine<br>Peters, Loraine H L; Maastricht University Care and Public Health Research Institute, Care and Public Health Research Institute (CAPHRI), Department of Family Medicine<br>van Schayck, Onno; Maastricht University, Care and Public Health Research Institute (CAPHRI), Department of Family Medicine<br>Burgers, Jako S.; Maastricht University, Care and Public Health Research Institute (CAPHRI), Department of Family Medicine<br>Ottenheijm, Ramon; Maastricht University, Care and Public Health Research Institute (CAPHRI), Department of Family Medicine<br>Gidding-Slok, Annerika; Maastricht University, Care and Public Health Research Institute (CAPHRI), Department of Family Medicine
**Primary Subject Heading**:	Patient-centred medicine
Secondary Subject Heading:	Qualitative research
Keywords:	Post-Acute COVID-19 Syndrome, Patient Reported Outcome Measures, Chronic Disease

SCHOLARONE™
Manuscripts

I, the Submitting Author has the right to grant and does grant on behalf of all authors of the Work (as defined in the below author licence), an exclusive licence and/or a non-exclusive licence for contributions from authors who are: i) UK Crown employees; ii) where BMJ has agreed a CC-BY licence shall apply, and/or iii) in accordance with the terms applicable for US Federal Government officers or employees acting as part of their official duties; on a worldwide, perpetual, irrevocable, royalty-free basis to BMJ Publishing Group Ltd ("BMJ") its licensees and where the relevant Journal is co-owned by BMJ to the co-owners of the Journal, to publish the Work in this journal and any other BMJ products and to exploit all rights, as set out in our licence.

The Submitting Author accepts and understands that any supply made under these terms is made by BMJ to the Submitting Author unless you are acting as an employee on behalf of your employer or a postgraduate student of an affiliated institution which is paying any applicable article publishing charge ("APC") for Open Access articles. Where the Submitting Author wishes to make the Work available on an Open Access basis (and intends to pay the relevant APC), the terms of reuse of such Open Access shall be governed by a Creative Commons licence – details of these licences and which Creative Commons licence will apply to this Work are set out in our licence referred to above.

Other than as permitted in any relevant BMJ Author's Self Archiving Policies, I confirm this Work has not been accepted for publication elsewhere, is not being considered for publication elsewhere and does not duplicate material already published. I confirm all authors consent to publication of this Work and authorise the granting of this licence.

For peer review only

Patient perspectives on the usability and content validity of the Assessment of Burden of Chronic Conditions (ABCC-) tool for post-COVID in the Netherlands: a qualitative study

Short title: ABCC-tool for Post-COVID

V.H.J. Debie[1], L.H.L. Peters[1], O.C.P. van Schayck[1], J.S. Burgers[1], R.P.G. Ottenheijm[1], A.H.M. Gidding-Slok[1]

[1] Department of Family Medicine, Care and Public Health Research Institute (CAPHRI), Maastricht University, Maastricht, the Netherlands.

Corresponding author:

Valerie Debie

P.O Box 616

6200 MD

Maastricht, the Netherlands

v.debie@maastrichtuniversity.nl

For peer review only

0031 88 388 7060

For peer review only

For peer review only - http://bmjopen.bmj.com/site/about/guidelines.xhtml

Abstract

Background: Post-COVID syndrome manifests with a diverse array of symptoms for which no standard care plan currently exists. Many questions were raised by patients, which underscored the need for a validated patient-reported outcome measure (PROM). Therefore, a post-COVID module was developed to be included in the Assessment of Burden Chronic Conditions tool (ABCC-) tool. The ABCC-tool evaluates and visualizes the perceived physical, emotional, and social burden of one or multiple chronic disease(s) using a balloon diagram, and aims to facilitate person-centered care and structured discussions between patients and healthcare professionals. This study explores the patients' perspective on the content of the ABCC-tool for post-COVID, and the tool's usability in a home-based setting.

Methods: All patients who completed the ABCC-tool for post-COVID were invited for an online semi-structured interview. We selected post-COVID patients who had used the tool in the past three months. Interviews were audio recorded and analyzed using a thematic approach with Atlas.ti version 23.

Results: Nineteen post-COVID patients (10 males, mean age 56) were interviewed between May and August 2024. The tool was regarded as user-friendly, and patients indicated they would use the tool again in the future. Patients valued the tool's broad range of topics, some of which are often overlooked in standard healthcare consultations. The tool was

For peer review only

comprehensible and relevant according to all patients. The balloon diagram was easy to understand, but a legend explaining the colors of the balloons was preferred. Other suggestions for improvement included adding open-text fields, periodic reminders to increase usability, and long-term results.

Conclusion: The ABCC-tool is a promising instrument for post-COVID patients, offering a structured way to monitor and communicate experienced burden in addition to standard healthcare consultations. Refinements addressing usability and comprehensiveness are recommended to facilitate its integration into clinical practices.

Keywords

Post-COVID, ABCC-tool, patient reported outcome measure, burden of disease

Strengths and limitations of this study

- The qualitative design provided in depth information about the content and patients' perspective of the ABCC-tool
- Data was analyzed using inductive and deductive approaches to increase the study's credibility

For peer review only

- Due to the lack of a clinical test for post-COVID, it was not possible to verify the patient's diagnosis

For peer review only

For peer review only - http://bmjopen.bmj.com/site/about/guidelines.xhtml

Introduction

Most people infected with SARS-CoV-2 (COVID-19) fully recover after the acute phase of the disease, regardless of the severity of their infection. However, according to the World Health Organization (WHO), six percent experience persistent symptoms that hinder their daily life activities, and decrease quality of life (QoL) [1-3]. Over 200 different symptoms have been reported, manifesting in various combinations. These may include, but are not limited to, respiratory, cognitive, and neurological symptoms, (chronic) fatigue, emotional burden, and work impairment [1,2,4]. This condition, known as post-COVID or long-COVID, is defined by the WHO as the emergence and persistence of these symptoms for at least two months, occurring three months after the initial infection, without any other identifiable cause [2].

Post-COVID patients reported persistent symptoms which raise many health-related questions, prompting them to visit their general practitioner (GP). Managing post-COVID is challenging due to the heterogeneity of symptoms and limited knowledge. To address these challenges, it is crucial that GPs take a comprehensive, holistic approach to patient care. This includes managing physical symptoms, supporting mental well-being and social participation, and monitoring recovery. Additionally, GPs could play a role in referring patients to specialists or rehabilitation programs when necessary. However, the absence of evidence-based

For peer review only - http://bmjopen.bmj.com/site/about/guidelines.xhtml

guidelines and the heterogeneous nature of symptoms make this task particularly demanding, and a supporting tool is deemed necessary [5-7].

Given the need for structured support, Lung Foundation Netherlands (an online Dutch forum and helpdesk for lung-related questions) decided in 2020 to develop a COVID-19-oriented tool to help patients with their concerns and coping with their symptoms. Due to the urgent need for patient support, it was decided to use already existing, validated, and well-known patient reported outcome measures (PROMs) as a foundation. Given that acute COVID-19 primarily affects the lungs, PROMs focused on pulmonary symptoms were assessed [8]. Eventually, the Assessment of Burden of Chronic obstructive pulmonary disease (ABC-) tool was selected and adjusted to create the Assessment of Burden of COVID-19 (ABCoV-) tool [8]. However, as the pandemic evolved, it became clear that a tool focusing solely on the acute phase of the disease provided only limited value to patients. Therefore, the ABCoV-tool was further adapted to serve as a post-COVID module within the Assessment of Burden of Chronic Conditions (ABCC-) tool.

As the successor to the ABC-tool, the ABCC-tool extends its focus beyond chronic obstructive pulmonary disease (COPD). Because of its modular format, every patient completes a set of generic questions, followed by one or more disease-specific modules. Until now, these disease-specific questions has been developed for asthma, COPD, type 2 diabetes mellitus,

For peer review only - http://bmjopen.bmj.com/site/about/guidelines.xhtml

chronic heart failure, osteoarthritis, cardiovascular risk management, and post-COVID [9-11]. The ABCC-tool was chosen because it measures burden of disease on a physical, emotional and social level, and because of its person-centered approach and stimulation of self-management and shared decision making. These are important factors for disease coping, improving health outcomes, and reducing the use of healthcare services [12]. The tool includes a self-administered questionnaire with 41 questions addressing physical, emotional, and social burden of disease, plus one open question, followed by a visual presentation of the results in the form of a balloon diagram (Figure 1) [9,11]. All questions are designed to be understandable at a B1 language level. This level indicates that patients can understand descriptions of events, feelings and wishes, and is characterized with short and easy sentences [13].

The ABCC-tool has been proven to increase perceived quality of care and patient activation [14]. A full description of the ABCC-tool and its intended use is published elsewhere [9].

For peer review only - http://bmjopen.bmj.com/site/about/guidelines.xhtml

[INSERT FIGURE 1 HERE]

Figure 1: The balloon diagram of the ABCC-tool for post-COVID. This diagram presents the results of the questionnaire from the ABCC-tool. Red balloons indicate a high burden of disease, while green balloons represent a low burden. Yellow and orange balloons depict intermediate scores, and grey balloons show the scores from the previous completion of the ABCC-tool.

In this study, we aimed to assess the content validity of the ABCC-tool for post-COVID and to evaluate its usability from the patients' perspectives. To the best of our knowledge, no other tool has the capability to measure physical, emotional, and social disease burden of post-COVID with the possibility to measure these disease burden in other chronic conditions, and visualize the results. Such tools are highly demanded by patients. Therefore, this evaluation was to ensure that the tool adequately covers all relevant content and to identify potential areas for improvement to make it easier for patients to use.

Methods

Study design

This qualitative study used an interpretative phenomenological approach to explore the experiences of post-COVID patients with the ABCC-tool in the home setting. This method

For peer review only

For peer review only - http://bmjopen.bmj.com/site/about/guidelines.xhtml

examines how individuals make sense of their experiences with the tool, and provides a structured framework for research design, data collection, and analysis, enabling a detailed exploration of their perspectives [15,16]. The COSMIN methodology for assessing the content validity of PROMs was used to assess the content validity [17]. The Consolidated Criteria for Reporting Qualitative Research (COREQ) checklist was used to ensure comprehensive and transparent reporting [18]. The full questionnaire is presented in Appendix 1.

Ethical approval was provided by the Medical Ethics Committee of the Maastricht University Medical Centre (METC 2022-3439). The study was funded by Pfizer and ZonMw, however, they did not have any involvement in the study design, data collection, analysis or manuscript writing process. All interviewed patients provided written informed consent before the interview.

Study participants

All patients who had used the ABCC-tool online via CuraVista (www.curavista.health) platform were invited for an online interview via email by CuraVista. CuraVista is an online eHealth platform for managing chronic diseases through monitoring and self-management programs. Patients can use this platform individually or together with their healthcare professional [19]. The aim was to interview fifteen to twenty patients until data saturation was achieved. Patients were eligible if they had a self-reported diagnosis of post-COVID according to the WHO-

For peer review only

For peer review only - http://bmjopen.bmj.com/site/about/guidelines.xhtml

definition, completed the ABCC-tool within the past three months to minimize recall bias, and were proficient in the Dutch language. Before the start of the interviews, patients were specifically asked whether they could remember completing the questionnaire and the balloon diagram, and whether they had post-COVID. Patients were excluded if they could not remember using the tool or seeing the balloon diagram. Patients were invited in small batches to account for potential non-responses and scheduling conflicts.

Data collection

Data were collected through single individual semi-structured online interviews in Dutch, conducted between May and August 2024 by one female researcher (VHJD), who was trained in qualitative research. All other researchers involved had experience in performing qualitive research. Relevant topics for the interview were outlined in a topic guide developed by the research team, which was pilot tested during the first interview. This topic guide was iteratively adjusted between interviews to address emerging themes to enhance data richness, such as the order of the questions. The final topic list is provided in Appendix 2. The two main topics included the content validity of the ABCC-tool and its usability. Prior to the interviews, patients received a printed copy of the tool's questionnaire and the balloon diagram by mail to recall the tool and facilitate discussion. All interviews were audio-recorded with patients' consent. No field notes were made during the interviews. The interviewer had no relationship with any of

For peer review only - http://bmjopen.bmj.com/site/about/guidelines.xhtml

the patients. Patients only were familiar with the aim of the research. The interviewer was introduced to the participants as a health scientist and researcher at Maastricht University.

Data analysis

Audio-recordings were subsequently transcribed using a custom implementation of WhisperX, which provides audio transcription based on Whisper (large-v2 model) and speaker diarization. This transcription process was performed locally on Maastricht University infrastructure/hardware [20]. Transcripts were 100% checked for correctness. Data analysis was conducted using a thematic approach with Atlas.ti version 23, software for qualitative data analysis. To analyze the transcripts, we used inductive – the process to identify new themes from textual data – and deductive – the process of applying pre-established codes to the transcripts – coding [21]. New themes were systematically monitored in between interviews to observe for data saturation and information power. Related codes were grouped into broader categories, and overarching themes were synthesized to gain a complete understanding of the patients' perspectives.

This iterative thematic analysis was reviewed and discussed with all members of the research team until consensus was reached. Initially, two researchers (VHJD and LHLP) independently coded 100% and 50% of all transcripts, respectively, to identify initial codes. The coding was

For peer review only

For peer review only - http://bmjopen.bmj.com/site/about/guidelines.xhtml

compared, and discrepancies were resolved through discussion, enhancing inter-rater reliability.

Patient and public involvement

No patients or public representatives were involved in any part of this study.

Results

In total, 5615 patients were invited for an interview, of which 27 interviews were started and 19 were completed and included in the analysis. Figure 2 presents the process of selecting and including patients. Ten men and nine women participated with a mean age of 56. Furthermore, approximately half of the patients had comorbidities. Eleven patients had a high educational level. No patients with a low educational level were included. In Table 1 the details per patient are presented. The length of the interviews ranged from 20 to 56 minutes. Data saturation was achieved for all topics, except comprehensiveness, after sixteen interviews. In no interview, additional people were present. One interview (interview 11) was cut short early due to concentration issues.

[INSERT FIGURE 2 HERE]

Figure 2: flowchart of the inclusion of patients for an interview

For peer review only

For peer review only - http://bmjopen.bmj.com/site/about/guidelines.xhtml

Table 1: Characteristics of the patients

Patient nr.	Age *	Gender	Other chronic conditions	Educational level**
1	Mid 50s	Female	Apnea<br>Lymphedema<br>Thyroid disorder	Medium
2	Late 50s	Female	-	High
3	Early 60s	Female	Asthma<br>Eczema	Medium
4	Early 50s	Female	Asthma	High
5	Early 60s	Male	Musculoskeletal disorder<br>Thyroid disorder	High
6	Mid 50s	Female	-	Medium
7	Late 50s	Female	-	High
8	Late 70s	Male	Apnea<br>Cardiovascular disease<br>Rheumatic disease	High
9	Early 50s	Male	Asthma	Medium
10	Early 50s	Male		Medium
11	Early 40s	Male	-	Medium
12	Mid 60s	Male	-	Medium
13	Late 50s	Female	-	High
14	Mid 50s	Male	COPD***	Medium
15	Late 40s	Male	-	High
16	Early 40s	Female	-	High
17	Early 50s	Male	-	High
18	Late 50s	Male	Rheumatic disease	High
19	Mid 60s	Female	-	High

*Age categories: early: age ending on 0-1-2-3, middle: 4-5-6, late: 7-8-9

**Dutch educational levels: *Low* = primary school and preparatory vocational secondary education (VMBO); *Medium* = senior general secondary education (HAVO), university preparatory education (VWO) and secondary vocational education and training (MBO); *High* = higher professional education (HBO) and research-oriented education (WO)[22]

*** COPD = chronic obstructive pulmonary disease

For peer review only

For peer review only - http://bmjopen.bmj.com/site/about/guidelines.xhtml

General evaluation of the ABCC-tool

Patients appreciated the tool's questionnaire and balloon diagram for providing a clear overview of both their current and past health status. The diversity of questions was also highly valued, especially those topics that were not typically linked to post-COVID by their healthcare professionals.

Patient 12: "I think it's a good tool. I believe it provides a lot of people with valuable insights into certain complaints, which might be related after all, things you wouldn't always immediately link, but that could indeed be connected in some way."

For peer review only

Several patients found it challenging to complete a questionnaire about their health over the past two weeks. They noted having both good and bad days, which made it difficult to answer the questions consistently. Some patients also struggled to remember their health status over the previous two weeks or from their last use of the tool. To address these challenges, some patients suggested incorporating extra text boxes to allow for personal notes.

Patient 13: "And what might be helpful […] is if there were spaces to write something down about your situation at that moment. […] For example, if I

look at my balloons now, I think, yeah, I have no idea what I was doing then, where I was, or if I was doing different things."

Most patients had used the ABCC-tool multiple times or expressed a willingness to use it more frequently. Nonetheless, many reported forgetting about the tool and suggested receiving periodic reminders, such as monthly or quarterly emails. Others mentioned they would only consider using the tool again if their symptoms changed.

Patient 11: "I think if I received an email saying it's been three months since you filled in the ABCC-tool. Click here to fill it in again, I would be more inclined to do it."

Seven patients required a more detailed explanation of how the tool worked, or what to do with it.

Patient 3: "I see the balloons, but I'm not really sure what I'm supposed to do with them."

Usability of the ABCC-tool

Not all patients could recall exactly where they found the ABCC-tool, but most patients had come across the ABCC-tool during online searches aimed at determining whether their symptoms and disease burden aligned with post-COVID. Others were alerted through

For peer review only - http://bmjopen.bmj.com/site/about/guidelines.xhtml

Facebook support groups, an email from C-support (a foundation of the Dutch ministry of Health, Welfare, and Sport that supports and advises people with post-COVID), or by chance through a Google search for treatments.

Patient 18: "C-support, I believe. I get a lot from them because once you're connected with them and that sort of things, you receive all kinds of information, so to speak."

Nobody experienced any difficulties with the practical use of the ABCC-tool. The website and login process did not present any problems either.

Patient 6: "No, I found it [the website] quite easy."

The aim of the ABCC-tool was not clear to everyone. Some patients thought it was part of a larger study to find an effective treatment for post-COVID, and that their participation would contribute to the development of new treatments or pharmaceutical interventions. However, most patients understood the aim of the tool.

Patient 13: "I thought it was also for a broader study. I didn't realize it was just for myself and possibly a healthcare provider. I actually didn't know that."

For peer review only - http://bmjopen.bmj.com/site/about/guidelines.xhtml

Most patients expressed a willingness to recommend the tool to other post-COVID patients, although some indicated they did not know any other post-COVID patients. One patient mentioned they would only recommend the tool if additional medical support would follow afterwards. Furthermore, two patients expressed a preference for more medical support after completing the ABCC-tool.

Patient 17: "It needs to be monitored. But if someone is consistently red month after month, then something is really wrong. And at that point, someone should be guided, like, hey, I think you need to be doing something else than what you're doing now. […] Or maybe referred to someone else."

Almost all patients found the questionnaire manageable in terms of length, as it could be paused and resumed at any time. However, one patient mentioned a lack of concentration to complete it and found the questionnaire too long.

Patient 7: "Yes, it was a nice experience. And I definitely liked that it can be saved in between. So, you don't have to complete it all at once. I did it in two parts."

For peer review only

For peer review only - http://bmjopen.bmj.com/site/about/guidelines.xhtml

Content of the ABCC-tool

The questions in the questionnaire were clear to all interviewed patients.

> *Patient 2: "Otherwise, I find it very clear. The language is very clear, and also how you rate [the answer from 0-6] it, so to speak, or the choices you have. Yes, it's just very clear to me."*

Regarding the comprehensiveness of the tool's questionnaire, data saturation was not achieved during the interviews. However, patients agreed that the most burdensome and most frequent burdens are included into the questionnaire. Additionally, four patients suggested including open-text fields throughout the questionnaire, allowing respondents to add personal notes about their current health status. This would facilitate easier comparison of responses across assessments. Another patient proposed incorporating a multiple-choice question to document any changes in areas such as health status, work, family situation, or other relevant personal factors.

Additionally, patients recommended adding questions regarding the type of therapy undergone, general pain experiences, balance difficulties, weight fluctuations due to edema, limitations in daily routines, symptom duration, feelings of being understood by government agencies, and restrictions experienced after intense physical activity.

For peer review only

275 Several suggestions were made to split questions that contain multiple items into separate questions. For example, two patients recommended breaking down the question on low, moderate, and vigorous physical activities into smaller steps to enable the possibility of more green balloons.

279 All questions were deemed relevant, except for one regarding sexuality, which was considered inappropriate by some in case of sharing the balloon diagram with their employer. Another patient felt this question was irrelevant for older individuals.

282 *Patient 18: "I can imagine that the topic of sexuality in such a chart... it's not very convenient if you want to show it to your boss. That's not something they should be involved in."*

285 The answer options were well-received, with most patients finding both the wording and number of response choices suitable.

287 *Patient 11: "I think 0 to 6 is good. Actually, very good."*

288 ***Visual representation***

289 Most patients found the colored balloons easy to understand and fun to use. Furthermore, the balloon diagram made it easier to provide the patient with helpful insights into their health status. However, some patients were confused about whether the height of the balloons (e.g.

For peer review only - http://bmjopen.bmj.com/site/about/guidelines.xhtml

low balloons indicating low burden) or the colors (e.g. green balloon indicating low burden) indicated the burden of disease or were uncertain about the grey balloons and required further clarification. Those who had difficulty understanding the balloons suggested adding a legend or marking 'high' and 'low' burdens of disease on the vertical axis. Another patient expressed interest in seeing more long-term data, e.g. results from the past six or seven assessments.

Patient 12: "At least putting something down like high-low or a lot or not. […] That would have been much clearer, yes."

All patients recognized their health status in the balloon representation. While some appreciated the simplicity of the balloons for providing a clear overview of their current situation, others found the abundance of red balloons confronting. Furthermore, patients varied in their level of acceptance regarding their situation, with some demonstrating greater acceptance than others.

Patient 2: "It was really confronting for me, but it was clear. You really see it there, that was very helpful for me, to really tell myself […] it's really real."

Some patients shared the balloon diagram with healthcare professionals to discuss their current and past health, although not all healthcare professionals expressed interest in the balloon diagram. Those who preferred to use the ABCC-tool and balloon diagram together with

For peer review only

a healthcare professional would like to use it during visits with GPs, physical therapists, occupational therapists, rehabilitation specialists, or company doctors. While not everyone considered discussing the diagram with a healthcare professional, some patients expressed an intention to do so in the future. Others shared the balloon diagram with family, friends, or colleagues to explain their experiences with post-COVID. However, some patients felt they did not need the diagram, as they were able to explain their condition effectively without it. One patient found the diagram too confronting to share with others.

Patient 1: "No, I never showed the balloons. But I do know where my stumbling blocks are and what I need to work on."

Patient 6: "Yes, with my family members, my children. [...] I find it nice to explain to them why I can't do certain things at times. Why I'm occasionally a bit down. [...] They also understand it better."

Discussion

This study aimed to assess patients' perspectives on using the ABCC-tool for post-COVID, and the content validity of the questionnaire within the tool. As patients are experiential experts in their disease, in-depth interviews with them provided meaningful insights, emphasizing

For peer review only - http://bmjopen.bmj.com/site/about/guidelines.xhtml

325 essential elements for the tool's use. The ABCC-tool was regarded as user-friendly and
326 provided a comprehensive overview of the post-COVID burden.

327 Findings from this study confirm that the tool includes the most commonly reported post-
328 COVID burdens and offers patients the opportunity to document additional concerns through
329 an open-text feature. Furthermore, patients appreciated the tool for multiple reasons, meaning
330 that the tool appears useful. First, the diversity of topics included in the tool, particularly as
331 many of these are not routinely addressed in their healthcare. Second, patients found the tool's
332 language accessible, because of its short sentences and simple wording [23]. Third, the length
333 of the questionnaire was acceptable. Fourth, the visualization gave an insightful overview of
334 disease burden. Finally, the tool was easy to find and use online.

335 For a few patients, the aim of the tool, and this study, was unclear and thought that the tool
336 was part of a larger study to find a therapy or cure for post-COVID. This highlights how easily
337 PROMs can be misunderstood, with potential consequences for engagement and
338 expectations. To prevent such misperceptions, it is important that future use of the ABCC-tool
339 is accompanied by clear communication about its purpose as a self-management and shared
340 decision-making instrument, rather than as a diagnostic or therapeutic tool.

341 Since the emergence of post-COVID, several PROMs have been developed and validated to
342 assess the disease burden, on functional status, symptom burden, QoL, and post-COVID

For peer review only - http://bmjopen.bmj.com/site/about/guidelines.xhtml

related stigma [24]. Examples of tools are the Symptom Burden Questionnaire for Long COVID (SBQ-LB), the Post COVID-19 Condition Stigma Questionnaire (PCCSQ), and the Long COVID Symptoms and Severity Score (LC-SSS scale) [25-28]. When comparing these tools to the ABCC-tool, we concluded that the ABCC-tool (44 items) is similar in length compared with the LC-SSS (44 items) and PCCSQ tool (40 items), with all three being scored on a 4 to 7 point Likert scale [25-28]. However, the ABCC-tool stands out, as none of these other tools specifically focus on the core dimensions of disease burden as defined by the WHO (i.e., physical, emotional, and social burden) and incorporate a visual representation of the results. It is important, however, to note that while these tools differ in scope and structure, no formal comparison was conducted regarding user experience or completion time. Therefore, although patients rated the ABCC-tool as user-friendly in this study, no conclusions can yet be drawn about its relative usability or efficiency compared to other available post-COVID PROMs. Future studies could explore these aspects in more depth through head-to-head comparisons.

In the current study, patients specifically preferred this visual representation. Although the balloon diagram was not immediately clear to everyone, a brief explanation made it fully understandable. Therefore, the inclusion of a legend for the balloon diagram was considered essential. This is congruent with recommendations that have been made in studies with the

For peer review only

For peer review only - http://bmjopen.bmj.com/site/about/guidelines.xhtml

ABC-, ABCC-, and Assessment of Burden of ColoRectal Cancer (ABCRC-) tool, to facilitate better understanding of the diagram [12,29,30]. The balloons gave patients understandable insights into their health status, facilitating structured discussions with healthcare professionals. However, some patients had numerous red balloons which felt confronting and negatively impacted on their motivation, for example, to adopt a healthier lifestyle. It is important to note that many of these patients had comorbidities, making it difficult to determine whether their reported burdens were due to post-COVID or these pre-existing conditions. Therefore, incorporating post-COVID into the ABCC-tool is more beneficial than developing a stand-alone tool, as the ABCC-tool includes multiple chronic conditions in a single PROM by integrating the relevant modules for each condition [9-11].

Additionally, other adjustments were recommended to make the tool more suitable for use, these include, amongst others, more open-text fields to accommodate personal notes. Given that memory loss and cognitive impairment are well-documented burdens of post-COVID (2), patients often struggle to recall their experiences from previous weeks. This leads to difficulties in distinguishing day-to-day changes. Therefore, patients preferred the option to make notes to ensure more accurate responses. Furthermore, both patients in this study and those utilizing the ABC-, ABCC-, and ABCRC-tool have suggested the implementation of periodic email reminders to encourage more frequent completion of the questionnaire [12,29,30]. Research

For peer review only

For peer review only - http://bmjopen.bmj.com/site/about/guidelines.xhtml

indicates that sending email reminders can significantly improve the completion rate of PROMs (22). Patients indicated that the tool would be more relevant if they had more frequent follow-up results to better monitor their progress.

Only limited evidence is available on optimal treatment strategies for post-COVID, as it is a relatively new disease. Currently, most treatment programs involve wait-and-see approaches, physical exercise, and lifestyle recommendations [31,32]. Ideally, post-COVID care should move towards a more personalized and holistic approach. However, this is challenging due to the lack of robust evidence on what interventions are effective per symptom, the high number of reported symptoms, and the substantial variability in burden among individuals [2,5,6]. Given these uncertainties, the ABCC-tool could play a role in addressing this gap.

Strengths and limitations

This study has several strengths and limitations. The qualitative study design provided in depth results in the content validation and the perspective of patient of the ABCC-tool. The use of both inductive and deductive approaches strengthened the study's credibility. The inductive coding process minimized confirmation bias by allowing themes and patterns to naturally emerge from the data, rather than being predefined. Meanwhile, deductive analysis reduced researcher bias by cross-verifying themes with existing frameworks [33-36].

For peer review only

For peer review only - http://bmjopen.bmj.com/site/about/guidelines.xhtml

Data saturation was not fully achieved on comprehensiveness, however, we systematically monitored whether new symptoms or themes emerged during the interviews. While some additional complaints were mentioned, these largely overlapped with issues already identified, or were not applicable for the ABCC-tool. We therefore argue that information power was sufficient, as the sample provided rich and relevant data on the clarity and relevance of the questionnaire items. Factors such as the study's aim and the quality of the collected data play an important role in determining whether the sample holds sufficient information for meaningful analysis [37]. Due to the complexity of post-COVID and the aforementioned over 200 symptoms that are associated with this condition [2], it is not feasible to include all symptoms in the questionnaire. Furthermore, the included symptoms reflect the most prevalent burdens described in the literature on post-COVID, such as fatigue and cognitive dysfunction, which were consistently confirmed by our participants [1,2,4]. We therefore decided that this issue can only be solved by incorporating an open question asking complaints other than those already mentioned. This way, the most prevalent burdens are included into the tool, with space for additional burdens to report.

In total, only 143 of the 5615 invited patients were eligible and interested in participating. We suspect that concentration problems commonly associated with post-COVID may have discouraged many patients from completing the interviews, as these were perceived as too

For peer review only - http://bmjopen.bmj.com/site/about/guidelines.xhtml

demanding, amongst others. This may have led to selection bias: those who volunteer to participate in scientific research are mostly woman, higher educated, and score higher on cognitive tests [38]. The underrepresentation of patients with a low educational level could potentially limit the external validity of the findings.

We interviewed a roughly equal number of men (n=10) and women (n=9), including patients with and without comorbidities. However, we were not able to take into account educational level when including patients, as this information was unavailable beforehand. Despite the random selection no participants with lower educational levels were included. Regardless, we expect the tool's questions to be accessible and readable for individuals with lower education due to their B1 language level. Furthermore, previous studies on the ABCC tool's content validity and patient experiences included participants with both higher and lower educational levels [10,12]. None of the patients reported difficulties with using the tool or understanding its language. Although the current study focused on a different patient population, the tool's purpose, functioning, and linguistic level remain unchanged. For future research, we recommend collecting basic patient information to enable more targeted recruitment and selection.

Our inclusion criteria were not objectively verifiable. First, we could not confirm whether the patient had post-COVID or not, as there is no standardized or validated diagnostic method for

For peer review only

For peer review only - http://bmjopen.bmj.com/site/about/guidelines.xhtml

this condition available. Currently, diagnoses are typically made by healthcare professionals or self-reported by patients, based on a set of symptoms. Second, it was impossible to verify the timing of patients' last use of the tool online, as these data were not accessible by the research team. To address these limitations, patients were asked at the start of the interview to specify who provided their post-COVID diagnosis and elaborate when this was self-diagnosed. They were also asked to indicate when they last used the ABCC-tool and whether they could remember this to minimize recall bias. If either of these two criteria was not met, the interview was terminated. For some patients, lack of concentration was a significant burden of post-COVID, which impacted their ability to participate in lengthy interviews. One interview had to be cut short on the demand of the patient. Additionally, some patients mentioned at the beginning of the interview not to be able to talk longer than a specific time. In that case, the interviewer started with the most important topics of the study (i.e. usability of the tool and content validation of the questionnaire). For the same reason, transcripts were not returned to the patients because this would be too burdensome. However, we acknowledge that offering participants the option to review their transcripts could have provided an additional opportunity for validation and feedback, and we will consider this approach in future studies.

For peer review only

449 *Implications for future research and practice*

450 A few adjustments (open text fields, periodic reminders, including a figure legend, and clarifying
451 the purpose of the tool) need to be made to the tool, based on the findings in this research.
452 Furthermore, although the questions of the tool have now been validated, a thorough
453 evaluation of the other aspects of the tool is in place. For example, the recommended score
454 calculation and lifestyle advice regarding physical activity might not be appropriate in the case
455 of post-exertional malaise (PEM). This needs further investigation.

456 After these adjustments, and further evaluation of the scores, a pilot study should be conducted
457 to evaluate the usability and feasibility of the tool in healthcare settings. This study should
458 include interviews with patients and healthcare professionals to evaluate the barriers and
459 facilitators for using the tool, and study the usability from different perspectives. Additionally,
460 evidence-based treatment recommendations for each domain must be developed and
461 integrated into the tool before clinical implementation. Technical aspects, such as seamless
462 integration into electronic health records, should also be addressed to ensure its practical use
463 in healthcare systems. These steps would enable the ABCC-tool to enhance care for post-
464 COVID patients.

465 Given the positive results of the ABCC-tool in assessing disease burden in patients with post-
466 COVID, further research is needed to explore its implementation in clinical practice. As post-

For peer review only - http://bmjopen.bmj.com/site/about/guidelines.xhtml

COVID is a relatively new condition, the first step is to establish guidelines supporting person-centered care and shared decision making. The ABCC-tool could play a key role in supporting this by providing a structured and efficient holistic assessment of patients' burden and needs.

Although the ABCC-tool was originally developed in the Netherlands, future research could explore its implementation in other countries. This would require careful translation and cultural validation to ensure that the tool is both linguistically accurate and contextually appropriate for use in different healthcare settings. The first interest in the ABCC-tool of other countries has been reported already. Furthermore, it may be valuable to explore the potential application of the ABCC-tool for post-COVID in patients with myalgic encephalomyelitis/chronic fatigue syndrome (ME/CFS), as the burden associated with ME/CFS closely resembles that of post-COVID. Both conditions are characterized by substantial health, economic, and social impacts, which are often underestimated due to limited awareness and understanding of these diseases [39]. In addition to the similarities in perceived disease burden, several studies have examined the prevalence of post-COVID and ME/CFS. These studies reported that 8–58% of participants met criteria for both post-COVID and ME/CFS [40-42].

For peer review only

For peer review only - http://bmjopen.bmj.com/site/about/guidelines.xhtml

Conclusion

The ABCC-tool is seen as a useful tool to assess experienced burden of disease in patients with post-COVID and to monitor the course of symptoms in time. The most appreciated aspects of the tool are the balloon diagram, which presents the current and past health situation of the patient, and the diverse range of questions in the questionnaire. Furthermore, the content of the questionnaire is praised for being comprehensive, comprehensible, and relevant for post-COVID. Based on the current and previous studies on the ABCC-tool, the tool for post-COVID holds significant potential to enhance person-centered care for post-COVID patients and is suitable for integration into clinical practice.

Declarations

Funding and competing interests

This study was funded by ZonMw (10430302130003) and Pfizer (75242181). The ZonMw grant was received by Jean Muris, with JSB as project leader. The grant of Pfizer was received by AHMGS. More information is available on https://www.pfizer.nl/. The funders had no role in study design, data collection and analysis, decision to publish, or preparation of the manuscript. All other authors have no completing interest to declare.

For peer review only

For peer review only - http://bmjopen.bmj.com/site/about/guidelines.xhtml

Authors' contributions

VHJD, RPGO, and AHMGS were involved in the study design. VHJD conducted the interviews. VHJD and LHLP performed the coding. All authors reviewed the thematic analysis and reached a consensus. Furthermore, all authors contributed to the writing process, provided feedback, and approved the final version of the manuscript. Guarantor is VHJD.

Ethics approval and consent to participate

Ethical approval was provided by the Medical Ethics Committee of the Maastricht University Medical Centre (METC 2022-3439). All interviewed patients provided written informed consent before the interview.

Availability of data and material

Data will be stored in a repository called DataHub. The DOI will become available shortly.

Acknowledgement

We would like to thank all the patients who showed interest in participating in this study. Furthermore, we would like to thank CuraVista for their contribution in the development and implementation of the ABCC-tool. In addition, this research was made possible, in part, using the Data Science Research Infrastructure (DSRI) hosted at Maastricht University.

For peer review only

References

1. Cha C, Baek G. Symptoms and management of long COVID: A scoping review. Journal of Clinical Nursing. 2024;33(1):11-28.
2. World Health Organization. Post COVID-19 condition (Long COVID) 2022. Available from: https://www.who.int/europe/news-room/fact-sheets/item/post-covid-19-condition
3. World Health Organization. Post COVID-19 condition (long COVID) 2025.
4. Frallonardo L, Ritacco AI, Amendolara A, et al. Long-Term Impairment of Working Ability in Subjects under 60 Years of Age Hospitalised for COVID-19 at 2 Years of Follow-Up: A Cross-Sectional Study. Viruses. 2024;16(5):688.
5. Bonilla H, Peluso MJ, Rodgers K, et al. Therapeutic trials for long COVID-19: A call to action from the interventions taskforce of the RECOVER initiative. Frontiers in immunology. 2023;14:1129459.
6. Greenhalgh T, Sivan M, Delaney B, et al. Long covid—an update for primary care. bmj. 2022;378.
7. Platteel TN, Koelmans JC, Cianci D, et al. Long-term prognosis of adults with moderately severe SARS-CoV-2 lower respiratory tract infection managed in primary care: Prospective cohort study. Eur J Gen Pract. 2025 Dec;31(1):2501306.
8. van Noort EMJ, Claessens D, Moor CC, et al. Online Tool for the Assessment of the Burden of COVID-19 in Patients: Development Study. JMIR Form Res. 2021 Mar 31;5(3):e22603.
9. Boudewijns EA, Claessens D, van Schayck OC, et al. ABC-tool reinvented: development of a disease-specific 'Assessment of Burden of Chronic Conditions (ABCC)-tool'for multiple chronic conditions. BMC family practice. 2020;21(1):1-7.
10. Debie VHJ, Boymans TAEJ, Ottenheijm RPG, et al. Expanding the ABCC-tool for Osteoarthritis: Development and Content Validation. Osteoarthritis and Cartilage Open. 2024;6(3):100488.
11. Slok AHM, Chavannes NH, van der Molen T, et al. Development of the Assessment of Burden of COPD tool: an integrated tool to measure the burden of COPD. NPJ Primary Care Respiratory Medicine. 2014;24(1):1-4.
12. Slok AH, Twellaar M, Jutbo L, et al. 'To use or not to use': a qualitative study to evaluate experiences of healthcare providers and patients with the assessment of burden of COPD (ABC) tool. npj Primary Care Respiratory Medicine. 2016;26(1):1-8.

For peer review only

For peer review only - http://bmjopen.bmj.com/site/about/guidelines.xhtml

547 13. Europass. Het gemeenschappelijk Europees referentiekader voor talen n.d. Available
548 from: https://europass.europa.eu/nl/common-european-framework-reference-
549 language-skills
550 14. Boudewijns EA, Claessens D, van Schayck OC, et al. Effectiveness of the Assessment
551 of Burden of Chronic Conditions (ABCC)-tool in patients with asthma, COPD, type 2
552 diabetes mellitus, and heart failure: A pragmatic clustered quasi-experimental study in
553 the Netherlands. European Journal of General Practice. 2024;30(1):2343364.
554 15. Tindall L. J.A. Smith, P. Flower and M. Larkin (2009), Interpretative Phenomenological
555 Analysis: Theory, Method and Research. Qualitative Research in Psychology. 2009
556 2009/11/25;6(4):346-347.
557 16. Pietkiewicz I, Smith JA. A practical guide to using interpretative phenomenological
558 analysis in qualitative research psychology. Psychological journal. 2014;20(1):7-14.
559 17. Terwee CB, Prinsen C, Chiarotto A, et al. COSMIN methodology for assessing the
560 content validity of PROMs–user manual. Amsterdam: VU University Medical Center.
561 2018.
562 18. Tong A, Sainsbury P, Craig J. Consolidated criteria for reporting qualitative research
563 (COREQ): a 32-item checklist for interviews and focus groups. Int J Qual Health Care.
564 2007 Dec;19(6):349-57.
565 19. van Zelst CM, Kasteleyn MJ, van Noort EMJ, et al. The impact of the involvement of a
566 healthcare professional on the usage of an eHealth platform: a retrospective
567 observational COPD study. Respir Res. 2021 Mar 21;22(1):88.
568 20. Radford A, Kim JW, Xu T, et al., editors. Robust speech recognition via large-scale
569 weak supervision. International conference on machine learning; 2023: PMLR.
570 21. Chandra Y, Shang L. Inductive Coding. In: Chandra Y, Shang L, editors. Qualitative
571 Research Using R: A Systematic Approach. Singapore: Springer Nature Singapore;
572 2019. p. 91-106.
573 22. CBS. Opleidingsniveau. Available from: https://www.cbs.nl/nl-
574 nl/nieuws/2019/33/verschil-levensverwachting-hoog-en-laagopgeleid-
575 groeit/opleidingsniveau
576 23. Ministerie van Algemene Zaken. Taalniveau B1. Available from:
577 https://www.communicatierijk.nl/vakkennis/rijkswebsites/aanbevolen-
578 richtlijnen/taalniveau-b1
579 24. Baalmann A-K, Blome C, Stoletzki N, et al. Patient-reported outcome measures for
580 post-COVID-19 condition: a systematic review of instruments and measurement
581 properties. BMJ Open. 2024;14(12):e084202.

For peer review only

25. Hughes SE, Haroon S, Subramanian A, et al. Development and validation of the symptom burden questionnaire for long covid (SBQ-LC): Rasch analysis. Bmj. 2022 Apr 27;377:e070230.
26. Damant RW, Rourke L, Cui Y, et al. Reliability and validity of the post COVID-19 condition stigma questionnaire: A prospective cohort study. EClinicalMedicine. 2023 Jan;55:101755.
27. Ye G, Zhu Y, Bao W, et al. The Long COVID Symptoms and Severity Score: Development, Validation, and Application. Value Health. 2024 Aug;27(8):1085-1091.
28. Tran VT, Riveros C, Clepier B, et al. Development and Validation of the Long Coronavirus Disease (COVID) Symptom and Impact Tools: A Set of Patient-Reported Instruments Constructed From Patients' Lived Experience. Clin Infect Dis. 2022 Jan 29;74(2):278-287.
29. Thomassen BJ, Gielen AH, Baak J, et al. Supporting follow-up care in colorectal cancer patients: first experiences with the Assessment of Burden of ColoRectal Cancer (ABCRC)-tool. Journal of Cancer Survivorship. 2024:1-11.
30. Peters LHL, Keijsers LCEM, van Schayck OCP, et al. Patient Experiences with the Assessment of Burden of Chronic Conditions (ABCC) Tool in Primary Care: A Qualitative Study. Submitted.
31. Bachmeier BE, Hölzle S, Gasser M, et al. How do German general practitioners manage long-/post-COVID? A qualitative study in primary care. Viruses. 2023;15(4):1016.
32. Kogel A, Machatschek M, Scharschmidt R, et al. Physical exercise as a treatment for persisting symptoms post-COVID infection: Review of ongoing studies and prospective randomized controlled training study. Clinical Research in Cardiology. 2023;112(11):1699-1709.
33. Fereday J, Muir-Cochrane E. Demonstrating rigor using thematic analysis: A hybrid approach of inductive and deductive coding and theme development. International journal of qualitative methods. 2006;5(1):80-92.
34. Hsieh H-F, Shannon SE. Three approaches to qualitative content analysis. Qualitative health research. 2005;15(9):1277-1288.
35. Nickerson RS. Confirmation bias: A ubiquitous phenomenon in many guises. Review of general psychology. 1998;2(2):175-220.
36. Nowell LS, Norris JM, White DE, et al. Thematic analysis: Striving to meet the trustworthiness criteria. International journal of qualitative methods. 2017;16(1):1609406917733847.

For peer review only

For peer review only - http://bmjopen.bmj.com/site/about/guidelines.xhtml

37. Malterud K, Siersma VD, Guassora AD. Sample size in qualitative interview studies: guided by information power. Qualitative health research. 2016;26(13):1753-1760.

38. Tripepi G, Jager KJ, Dekker FW, et al. Selection bias and information bias in clinical research. Nephron Clinical Practice. 2010;115(2):c94-c99.

39. Vester P, Boudouroglou-Walter S, Schreyögg J, et al. Burden of Disease in Myalgic Encephalomyelitis/Chronic Fatigue Syndrome (ME/CFS): A Scoping Review. Appl Health Econ Health Policy. 2025 Sep 23.

40. Dehlia A, Guthridge MA. The persistence of myalgic encephalomyelitis/chronic fatigue syndrome (ME/CFS) after SARS-CoV-2 infection: A systematic review and meta-analysis. Journal of Infection. 2024 2024/12/01/;89(6):106297.

41. AlMuhaissen S, Abu Libdeh A, ElKhatib Y, et al. Myalgic encephalomyelitis/chronic fatigue syndrome (ME/CFS) and COVID-19: is there a connection? Curr Med Res Opin. 2023 Aug;39(8):1119-1126.

42. Jason LA, Dorri JA. ME/CFS and Post-Exertional Malaise among Patients with Long COVID. Neurol Int. 2022 Dec 20;15(1):1-11.

For peer review only

For peer review only - http://bmjopen.bmj.com/site/about/guidelines.xhtml

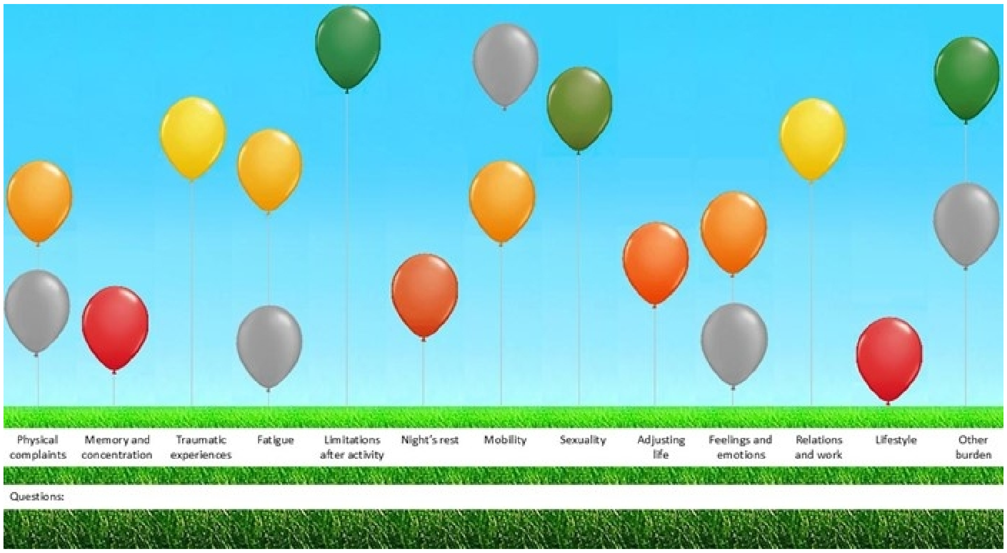

Figure 1: The balloon diagram of the ABCC-tool for post-COVID. This diagram presents the results of the questionnaire from the ABCC-tool. Red balloons indicate a high burden of disease, while green balloons represent a low burden. Yellow and orange balloons depict intermediate scores, and grey balloons show the scores from the previous completion of the ABCC-tool.

185x101mm (96 x 96 DPI)

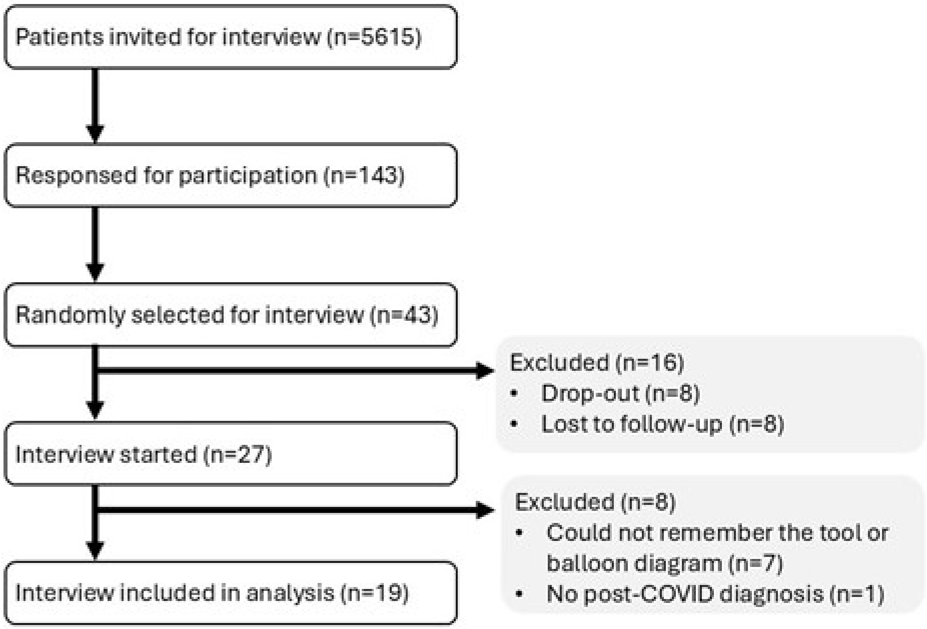

Figure 2: flowchart of the inclusion of patients for an interview

127x85mm (96 x 96 DPI)

APPENDIX 1: QUESTIONNAIRE IN THE ABCC-TOOL

ENGLISH VERSION

(Note: this is an unvalidated translation from Dutch to English specifically for this paper)

Dear Sir/Madam,<br>With this questionnaire, we would like to get an impression of how you are doing. During consultation with your healthcare provider, you can talk about the topics that are important to you.<br>These questions are related to the chronic condition(s) for which you are visiting the healthcare provider.

What were you suffering from?

	In the past week, how often ….	0 Never	1 Hardly ever	2 A few times	3 Several times	4 Many times	5 A great many times	6 Almost all the time
1	did you suffer from **fatigue**?	☐	☐	☐	☐	☐	☐	☐
2	did you have a poor **night's rest**?	☐	☐	☐	☐	☐	☐	☐
3	did you suffer from sadness, fear, frustration, shame or other **unpleasant feelings**?	☐	☐	☐	☐	☐	☐	☐
4	did you suffer **adjusting your life** (e.g. planning of activities, get enough exercise, eating healthy)?	☐	☐	☐	☐	☐	☐	☐
	In the past 2 weeks, to what extend …	0 Not at all	1 Very slightly	2 Slightly	3 Moderate	4 Very	5 Extremely	6 Totally
5	did you suffer from a **decreased condition**?	☐	☐	☐	☐	☐	☐	☐
6	did you suffer from **pain/ difficulties with breathing**?	☐	☐	☐	☐	☐	☐	☐
7	did you suffer from **coughing**?	☐	☐	☐	☐	☐	☐	☐
8	did you suffer from **pain on the chest** or **a feeling of pressure on the chest**?	☐	☐	☐	☐	☐	☐	☐
9	did you suffer from **palpitations**?	☐	☐	☐	☐	☐	☐	☐
10	did you suffer from a **strange taste or smell, or tasted/smelled nothing**?	☐	☐	☐	☐	☐	☐	☐
11	did you suffer from **earache** or **tinnitus**?	☐	☐	☐	☐	☐	☐	☐
12	did you suffer from a **lump in your throat** or a **sore throat**?	☐	☐	☐	☐	☐	☐	☐
13	did you suffer from problems with **chewing and/or swallowing**?	☐	☐	☐	☐	☐	☐	☐
14	did you suffer from **heavy arms or legs**?	☐	☐	☐	☐	☐	☐	☐
15	did you suffer from **joint and/or muscle pain**?	☐	☐	☐	☐	☐	☐	☐
16	did you suffer from **tingling in your hands, feet, arms or legs**?	☐	☐	☐	☐	☐	☐	☐
17	did you suffer from **a headache**?	☐	☐	☐	☐	☐	☐	☐
18	did you suffer from **dizziness**?	☐	☐	☐	☐	☐	☐	☐
19	did you suffer from problems with **concentration and/or memory**?	☐	☐	☐	☐	☐	☐	☐

	In the past 2 weeks, to what extend …	0 Not at all	1 Very slightly	2 Slightly	3 Moderate	4 Very	5 Extremely	6 Totally
20	did you feel **overstimulated**?	□	□	□	□	□	□	□
21	did you suffer from **bad memories, flashbacks or nightmares**?	□	□	□	□	□	□	□
22	did you suffer from **blurred and/or double vision, dry or painful eyes**?	□	□	□	□	□	□	□
23	did you suffer from **abdominal complaints**?	□	□	□	□	□	□	□
24	did you suffer from **feeling hot and cold alternately** or did you have a **fever**?	□	□	□	□	□	□	□
25	did you suffer from **dry or sore skin**?	□	□	□	□	□	□	□
26	did you suffer from **hair loss**?	□	□	□	□	□	□	□
	In the past 2 weeks, …	Not	2-3 hours	4-10 hours	11-13 hours	14-24 hours	24-48 hours	Longer than 48 hours
27	if you **felt worse after exercising**, how long did this last?	□	□	□	□	□	□	□
28	did you suffer from **any complaints other than those mentioned above**?	I suffered from:						

In what did you feel limited?

	In the past week, to what extend …	0 Not at all	1 Very slightly	2 Slightly	3 Moderate	4 Very	5 Extremely	6 Totally
29	were you limited in **strenuous physical** activities (such as climbing stairs, hurrying, doing sports)?	□	□	□	□	□	□	□
30	were you limited in **moderate physical** activities (such as walking, housework, carrying things)?	□	□	□	□	□	□	□
31	were you limited in **daily activities** at home (such as dressing, washing yourself)	□	□	□	□	□	□	□
32	were you limited in your **work** (paid word, unpaid work, volunteer work)?	□	□	□	□	□	□	□
33	were you limited in **moving around outside your home**?	□	□	□	□	□	□	□
34	Were you limited in your **social activities** (visiting friends and family, daytrips)?	□	□	□	□	□	□	□
35	had your condition a negative impact on your **relations with others**?	□	□	□	□	□	□	□
36	did you have any difficulty with **intimacy or sexuality**?	□	□	□	□	□	□	□
37	did you **worry about your future**?	□	□	□	□	□	□	□

For peer review only

How was your lifestyle?

38	In the past week, how many days have you had moderately intense **physical exercise** for 30 minutes or more?<br>E.g. walking or cycling in a fast pace<br>It may also be a minimum of 3x10 minutes	□ 0 days<br>□ 1-2 days<br>□ 3-4 days<br>□ 5 days or more
39	How many glasses of **alcohol** did you drink in the past week?	.... glasses per week
40	Do you **smoke** or have you smoked?	□ Yes. In the last week, how many (shag) cigarettes have you smoked on average per day? ____________<br>□ Previously. Stopped smoking since: ____ (month) / ____ (year)<br>□ Never
41	What is your **weight**?	.... kg
42	What is your **height**?	.... cm
43	In the past week, how many days did you **eat healthy**?	□ 0 days<br>□ 1-2 days<br>□ 3-4 days<br>□ 5 days or more
44	Is there anything else you would like to **discuss** or would like to receive more **information** about?<br>____<br>____	

For peer review only

DUTCH VERSION

Beste meneer/mevrouw,
Met deze vragenlijst willen we samen met u in kaart brengen hoe het met u gaat.
Deze vragen hebben te maken met de chronische aandoening(en) waarvoor u bij de zorgverlener komt.

Waar had u last van?

	In de afgelopen week, hoe vaak …	0 Nooit	1 Zelden	2 Af en toe	3 Regel-matig	4 Heel vaak	5 Meestal	6 Altijd
1	had u last van **vermoeidheid**?	☐	☐	☐	☐	☐	☐	☐
2	had u een slechte **nachtrust**?	☐	☐	☐	☐	☐	☐	☐
3	had u last van somberheid, angst, frustratie, schaamte of andere **vervelende gevoelens**?	☐	☐	☐	☐	☐	☐	☐
4	had u **moeite om uw leven aan te passen** (bijv. plannen van activiteiten, voldoende beweging, gezond eten)?	☐	☐	☐	☐	☐	☐	☐

	In de afgelopen 2 weken, in welke mate …	0 Hele-maal niet	1 Heel weinig	2 Een beetje	3 Tamelijk	4 Erg	5 Heel erg	6 Volledig
5	had u last van een **verminderde conditie**?	☐	☐	☐	☐	☐	☐	☐
6	had u last van **pijn of moeite met ademhalen**?	☐	☐	☐	☐	☐	☐	☐
7	had u last van **hoesten**?	☐	☐	☐	☐	☐	☐	☐
8	had u last van **pijn op de borst of een drukkend gevoel op de borst**?	☐	☐	☐	☐	☐	☐	☐
9	had u last van **hartkloppingen**?	☐	☐	☐	☐	☐	☐	☐
10	had u last van een **vreemde smaak of geur, of proefde/rook u niets**?	☐	☐	☐	☐	☐	☐	☐
11	had u last van **oorpijn of oorsuizen**?	☐	☐	☐	☐	☐	☐	☐
12	had u last van een **brok in uw keel of keelpijn**?	☐	☐	☐	☐	☐	☐	☐
13	had u last van **kauw- en/of slikproblemen**?	☐	☐	☐	☐	☐	☐	☐
14	had u last van **zware armen of benen**?	☐	☐	☐	☐	☐	☐	☐
15	had u last van **gewrichts- en/of spierpijn**?	☐	☐	☐	☐	☐	☐	☐
16	had u last van **tintelingen in uw handen, voeten, armen of benen**?	☐	☐	☐	☐	☐	☐	☐
17	had u last van **hoofdpijn**?	☐	☐	☐	☐	☐	☐	☐
18	had u last van **duizeligheid**?	☐	☐	☐	☐	☐	☐	☐
19	had u last van **concentratie- en/of geheugenproblemen**?	☐	☐	☐	☐	☐	☐	☐
20	had u het gevoel **overprikkeld** te zijn?	☐	☐	☐	☐	☐	☐	☐
21	had u last van **nare herinneringen, herbelevingen of nachtmerries**?	☐	☐	☐	☐	☐	☐	☐
22	had u last van **wazig en/of dubbelzien, droge of pijnlijke ogen**?	☐	☐	☐	☐	☐	☐	☐

	In de afgelopen <u>2 weken</u>, in welke mate …	0<br>Hele-maal niet	1<br>Heel weinig	2<br>Een beetje	3<br>Tamelijk	4<br>Erg	5<br>Heel erg	6<br>Volledig
23	had u last van **buikklachten**?	☐	☐	☐	☐	☐	☐	☐
24	had u last van u zich **afwisselend warm en koud voelen of had u koorts**?	☐	☐	☐	☐	☐	☐	☐
25	had u last van een **droge of pijnlijke huid**?	☐	☐	☐	☐	☐	☐	☐
26	had u last van **haaruitval**?	☐	☐	☐	☐	☐	☐	☐
	In de afgelopen <u>2 weken</u> …	Niet	2-3 uur	4-10 uur	11-13 uur	14-24 uur	24-48 uur	langer dan 48 uur
27	als u zich **slechter voelde na het doen van een inspanning**, hoe lang hield dat dan aan?	☐	☐	☐	☐	☐	☐	☐
28	had u last van **klachten anders dan die hierboven al genoemd zijn**?	Ik had last van:						

Waarin voelde u zich beperkt?

	In de afgelopen <u>week</u>, in welke mate …	0<br>Hele-maal niet	1<br>Heel weinig	2<br>Een beetje	3<br>Tamelijk	4<br>Erg	5<br>Heel erg	6<br>Volledig
29	voelde u zich beperkt in **zware lichamelijke activiteiten** (trap lopen, haasten, sporten)?	☐	☐	☐	☐	☐	☐	☐
30	voelde u zich beperkt in **matige lichamelijke activiteiten** (wandelen, huishoudelijk werk, boodschappen doen)?	☐	☐	☐	☐	☐	☐	☐
31	voelde u zich beperkt in **dagelijkse activiteiten** (u zelf aankleden, wassen)?	☐	☐	☐	☐	☐	☐	☐
32	voelde u zich beperkt in uw **werk** (betaald werk, onbetaald werk, vrijwilligerswerk)?	☐	☐	☐	☐	☐	☐	☐
33	voelde u zich beperkt in het u **verplaatsen buitenshuis**?	☐	☐	☐	☐	☐	☐	☐
34	voelde u zich beperkt in uw **sociale activiteiten** (uitjes, vrienden en familie bezoeken)?	☐	☐	☐	☐	☐	☐	☐
35	had uw aandoening een negatieve invloed op uw **relatie met anderen**?	☐	☐	☐	☐	☐	☐	☐
36	had u moeite met **intimiteit** of **seksualiteit**?	☐	☐	☐	☐	☐	☐	☐
37	maakte u zich **zorgen over uw toekomst**?	☐	☐	☐	☐	☐	☐	☐

For peer review only

Hoe ging het met uw leefstijl?

38	In de afgelopen week, hoeveel dagen heeft u 30 minuten of meer matig intensieve **lichaamsbeweging** gehad?<br>Bijv. stevig doorwandelen of harder fietsen.<br>Het mogen ook minimaal 3x10 minuten zijn.	□ 0 dagen<br>□ 1-2 dagen<br>□ 3-4 dagen<br>□ 5 dagen of meer
39	Hoeveel glazen **alcohol** dronk u in de afgelopen week?	… glazen per week
40	Rookt u of heeft u **gerookt**?	□ Ja. Hoeveel (shag)sigaretten heeft u in de afgelopen week gemiddeld per dag gerookt? ___________<br>□ Vroeger. Gestopt met roken sinds: ___(maand) / ___(jaar)<br>□ Nooit
41	Wat is uw **gewicht**?	……….. kg
42	Wat is uw **lengte**?	……….. cm
43	In de afgelopen week, hoeveel dagen heeft u **gezond gegeten**?	□ 0 dagen<br>□ 1-2 dagen<br>□ 3-4 dagen<br>□ 5 dagen of meer
44	Is er nog iets wat u wilt **bespreken** of waar u **meer informatie** over wilt krijgen?<br>____<br>______________________________________	

For peer review only

APPENDIX 2: TOPIC LIST (ORIGINALLY IN DUTCH)

Demographic questions

1. What is your age?
2. How is your health now?
    a. Do you have other chronic conditions?
3. How was your health before you had post-COVID?
4. What is your educational level?
5. What is your gender?

Acute COVID-19

6. When did you have acute COVID-19?
7. Were you admitted to the hospital?
    a. If yes: where you admitted to the intensive care?
8. How did you get the diagnosis?

Post-COVID

9. Since when do you have post-COVID symptoms?
10. By whom and how where you diagnosed with post-COVID

ABCC-tool

11. How did you discover the ABCC-tool?
12. When and how many times did you complete the ABCC-tool?
13. Would you like to use the ABCC-tool again in the future?
14. Why did you complete the ABCC-tool?
    a. What was your purpose with it?
    b. Did the ABCC-tool meet those purposes?

Content validation

15. Was the ABCC-tool complete (comprehensive)?
16. Was question ... relevant for you? (per item in the questionnaire)
17. Was question ... formulated clearly? (per item in the questionnaire)
18. What were your thoughts about the answer options?

Usability

19. How was the ABCC-tool online to use?
    a. Was it easy to log in on the app/website?
    b. Was the app/website clear?
20. How much time did it take?
21. What were your thoughts about the balloon diagram?
    a. Was it clear?
    b. Would you like to change anything about it?
22. Did you discuss the balloon diagram with somebody?
    a. If yes: with whom? And why?
23. In what way has the balloon diagram helped you explain how you feel?
    a. Did it give you more compassion from a healthcare professional?
    b. Did it give you more compassion from family or friends?
24. Would you recommend the ABCC-tool to others?
    a. If yes: to whom do you want to recommend the ABCC-tool?

For peer review only
